# Trimmed *N*-glycans define aggressive gastric cancer and predict clinical outcomes

**DOI:** 10.7150/thno.111670

**Published:** 2025-08-22

**Authors:** Dylan Ferreira, Beatriz Marinho-Santos, Marta Relvas-Santos, Bernardo Orr, Andreia Brandão, Luís Pedro Afonso, Lúcio Lara Santos, José Alexandre Ferreira

**Affiliations:** 1 Research Center of IPO-Porto (CI-IPOP) / RISE@CI-IPOP (Health Research Network), Portuguese Oncology Institute of Porto (IPO-Porto) / Porto Comprehensive Cancer Center (P.ccc) Raquel Seruca, Porto, Portugal.; 2 School of Medicine and Biomedical Sciences (ICBAS), University of Porto, Porto, Portugal.; 3 School of Medicine and Biomedical Sciences of University Fernando Pessoa, Porto, Portugal.; 4 Department of Pathology, Portuguese Oncology Institute of Porto (IPO-Porto), Porto, Portugal.; 5 Department of Surgical Oncology, Portuguese Oncology Institute of Porto (IPO-Porto), Porto, Portugal.; 6 GlycoMatters Biotech, 4500-162, Espinho, Portugal.

**Keywords:** cancer glycobiology, precision oncology, gastric cancer, glycoproteome, glycome

## Abstract

**Rationale:** Gastric cancer (GC) is a leading cause of cancer-related mortality, particularly in advanced stages where prognosis and targeted treatment remain challenging. The glycocalyx, a dense network of glycans and glycoproteins, is critical for tumor progression and immune evasion, yet its molecular signatures are poorly understood. This study investigates glycan-based biomarkers of aggressiveness, focusing on paucimannosidic *N*-glycans, a previously underexplored glycosylation pattern in cancer.

**Methods:** High-throughput *N*-glycome analysis was performed on gastric tumors of varying aggressiveness, followed by *Galanthus Nivalis* Lectin (GNL) immunostaining to assess paucimannosidic glycans across tumor stages. Comparative analysis was performed against clinically relevant GC biomarkers (E-cadherin, p53, MSI, sTn, sLeA). TCGA analysis correlated key paucimannose-associated glycosyltransferases with clinical outcomes. Glycoproteomics identified glycoproteins carrying paucimannoses, later validated using immunoassays in tumor tissues for clinical relevance. Additionally, serum samples were analyzed to evaluate the non-invasive potential of GNL reactivity and associated glycoproteins.

**Results:** Aggressive gastric tumors were significantly enriched in paucimannosidic *N*-glycans, a feature not previously reported in this malignancy. Lectin immunoblotting confirmed their disease specificity, with expression increasing with tumor progression. GNL staining outperformed established biomarkers in prognostic accuracy. TCGA analysis of more than 400 cases showed a strong correlation between high paucimannose-associated glycosyltransferase expression and poor prognosis. Glycoproteomics unexpectedly revealed paucimannose *N*-glycans primarily on intracellular ribosomal proteins, though key membrane proteins like MMP9 displayed aberrant paucimannosylation. MMP9 expression increased with tumor stage and grade, with tumors co-expressing MMP9 and paucimannosidic glycans exhibiting the worst prognosis. In serum, only MMP9 demonstrated diagnostic potential as a circulating biomarker, whereas GNL did not show a significant association.

**Conclusions:** This study provides the first comprehensive characterization of the GC glycome, linking paucimannosidic *N*-glycans to tumor aggressiveness and poor clinical outcome. These glycans demonstrated superior prognostic performance compared to established clinical biomarkers. Their association with MMP9 further suggests a key role in disease progression. Together, these findings suggest that alterations in *N*-glycosylation, including paucimannosylated glycoproteins, hold promise for future prognostic and therapeutic applications in gastric cancer.

## Introduction

Gastric cancer (GC) remains a significant global health challenge due to its aggressive progression and poor prognosis, particularly in advanced stages 1, 2. This underscores the critical need for reliable biomarkers to support accurate patient stratification and targeted therapeutic interventions.

The glycocalyx, a dense layer of glycoproteins, glycolipids, and glycosaminoglycans on the cell surface, plays crucial roles in cell signaling, adhesion, and protection against microenvironmental stress 3, 4. Notably, aberrant glycosylation is now recognized as a hallmark of cancer, driving tumor progression through dysregulated receptor signaling, immune evasion, and metastatic dissemination, while offering valuable insights for clinical decision-making 5-7. Furthermore, alterations in glycosylation often lead to disease-specific glycoproteoforms that are localized on the cell surface, holding potential as therapeutic targets 8-12. Namely, it has long been established that advanced tumours overexpress short glycans such as the Tn and sialyl-Tn (sTn) antigens, resulting from premature truncation of mucin-type *O*-glycosylation 13. These aberrant *O*-glycans contribute to tumour progression by disrupting cell-cell adhesion and promoting immune evasion through interactions with inhibitory receptors on immune cells 14-16. More recently, growing evidence has highlighted that *N*-glycans, typically known for their elongated and branched structures, can also exist in truncated forms, such as paucimannose-type glycans 17. Paucimannoses, are a subset of trimmed glycans, are simplified *N*-glycans composed of 1-3 mannose residues attached to the conserved GlcNAc₂ core, with or without a fucose residue. These glycans arise due to altered activity of Golgi mannosidases and deficient elongation by *N*-acetylglucosaminyltransferases, leading to stalled glycan maturation 17-19. Unlike complex *N*-glycans, trimmed glycans such as paucimannoses lack extensive branching and terminal decorations, reflecting incomplete glycosylation processes 20, 21. Paucimannoses have been observed in various tumor types, including colorectal 22, renal 23, lung 23, and glioblastoma cancers 24. High levels of paucimannosylation correlate with poor prognosis in advanced colorectal cancer 25. Functionally, paucimannose-type *N*-glycans are recognized by innate immune lectins such as DC-SIGN and the mannose receptor, which can trigger tolerogenic signaling in dendritic cells and macrophages, contributing to immune suppression 19, 23, 26. Additionally, these glycans influence the trafficking and stability of surface proteins like integrins and growth factor receptors, promoting cell migration, invasion, and increased resistance to apoptotic cues.

In GC, specific glycosylation changes have been identified as key drivers of cancer progression. These include immature Tn and sTn antigens, as well as oversialylation found in more extended *N*- and *O*-glycans found in other solid tumours 13, 27. These modifications enhance tumor cell adhesion, migration, immune evasion, and activate oncogenic pathways such as c-Met, RON, and ErbB receptor signaling 28-30. Furthermore, these glycan changes are associated with poor prognosis, with some, like sialyl Lewis A (CA19-9) and sTn (CA72-4), already used as serological biomarkers for clinical monitoring of disease and recurrence 31-34. Notably, while alterations in *O*-glycosylation have been widely reported in GC, the *N*-glycome remains comparatively less explored, with many aspects of its functional relevance still unclear. Nevertheless, emerging glycomics studies have begun to shed light on specific alterations, such as the increased expression of branched *N*-glycans, which are closely associated with enhanced tumour invasiveness and metastatic potential 35-39. These structures are frequently terminated with sialic acids, giving rise to sialylated Lewis antigens, which mediate metastasis and contribute to GC's aggressiveness 29, 35. Additionally, core-fucosylated *N*-glycans have been found to be downregulated in GC, with studies suggesting that their restoration may inhibit tumour cell proliferation 40, 41. However, the complexity of GC's glycome is exemplified by contrasting reports on *N*-glycan alterations. While earlier studies describe increased expression of bisecting GlcNAc structures, recent research indicates the opposite, with their overexpression potentially exerting tumour suppressive effects 29, 38, 42, 43. Also, immature *N*-glycan structures such as paucimannoses remain virtually uncharacterized in GC, despite being increasingly recognized in other tumour types. These findings underscore a critical gap in our understanding of the *N*-glycome in GC. Despite growing evidence of glycan alterations, most studies have focused on isolated glycan classes or relied on single analytical approaches, failing to capture the full complexity and dynamic nature of glycosylation 44. As a result, the contribution of the glycome to cancer progression and metastasis across disease stages remains largely unresolved. Addressing this requires systematic and integrative glycomic profiling. Equally important is the characterization of the glycoproteome, as the functional impact of glycosylation depends on the specific proteins modified and their cellular context. Glycoproteomics enables the identification of proteins carrying aberrant glycosylation and allows site-specific mapping of glycosylation patterns, revealing functionally relevant glycoproteoforms linked to tumour biology. Integrating glycomic and glycoproteomic data can improve biomarker specificity and therapeutic targeting, paving the way for more precise strategies in gastric cancer 44, 45. Addressing this gap, we aim to provide a comprehensive characterization of the GC *N*-glycome and glycoproteome, focusing on their evolution during disease progression. By enhancing our understanding of glycocalyx alterations, we seek to uncover molecular insights that improve patient stratification and inform the development of novel therapeutic strategies for GC.

## Methods

### Patient sample set

Galanthus Nivalis Lectin (GNL) reactivity, E-cadherin, Sialyl-Tn (sTn), Sialyl-Lewis A (sLeA), p53 protein and Microsatellite Instability (MSI) were retrospectively analysed in 148 formalin-fixed paraffin-embedded (FFPE) primary gastric tumour tissues obtained from the IPO-Porto biobank, reflecting two distinct clinical stages [early-stage (I/II) versus advanced-stage (III/IV) GC]. Early-stage cases were typically characterized by localized disease (T1-T2, N0/N1, M0), while advanced-stage includes cases with deeper invasion (≥ T3), lymph node involvement (≥ N2), or distant metastasis (M1), following the American Joint Committee on Cancer 8th edition criteria 46. Moreover, cases were classified according with the size of the tumor (T), the involvement of nearby lymph nodes (N), and the presence of distant metastasis (M), based on TNM classification system 46. The patient sample set included 66 female and 82 male patients, aged between 34 and 89 years (average age: 64 years), who underwent gastrectomy at IPO-Porto from 2004 to 2016. A subset of 30 out of 148 FFPE primary gastric tumour tissues was selected for analysis of MMP9 expression levels. Additionally, both MMP9 and GNL staining were analysed in 10 FFPE lymph nodes metastases in an independent sample set of 5 male and 5 female patients, aged between 39 and 85 years (median age: 56 years). All clinicopathological information used to assess clinical relevance is summarized in** Table 1, 2 and 3**. Clinicopathological data were extracted from the patients' medical records. This study received approval from the IPO institutional Ethics Committee (Approval No. CES 87/017) and was conducted following informed written consent from all participating patients. All study methodologies were performed according to the standards set by the declaration of Helsinki. A series of healthy tissues recovered from autopsies, including colon, pancreas, stomach, skin, testis, spleen and thyroid were also screened for these antigens to assess cancer specificity.

### Mass spectrometry-assisted *N*-glycomics

*N*-glycomics was performed on 13 FFPE GC tissue samples based on methods previously described by Zhang T and Madunic K *et al*. 47. Samples for analysis were elected by a certified pathologist after confirming tumor content and excluding necrotic or poorly preserved areas. Clinicopathological data of patients included in the *N*-glycomics analysis is summarized in **Table 4**. Briefly, FFPE tissues were deparaffinized, rehydrated, and subjected to heat-induced antigen retrieval using a citrate-based solution (Vector Laboratories). Then, proteins were denatured and reduced by incubation with 150 µL denaturation mix [145 µL of 8 M GuHCl and 5 µL of 200 mM dithiothreitol (DTT)] at 60 °C for 30 min. *N-*glycan release was achieved after digestion with PNGase F (1 U/10 µg protein at 37 °C overnight; Promega). Released *N*-glycans were hydrolyzed with 25 µL of 100 mM ammonium acetate at pH 5 (1 h at RT), removing the glycosylamine form of the non-reducing terminus, and subsequently reduced with 20 µL of 1 M NaBH_4_ in 50 mM KOH (3 h at 50 °C). The reaction was quenched by adding glacial acetic acid. Then, *N*-glycan samples were desalted using a cation exchange resin (AG 50W-X8; Bio-Rad). Finally, reduced *N*-glycans were permethylated and analyzed by reverse phase nanoLC-ESI-MS/MS as previously described by us 48, using a Vanquish neoUHPLC nano-LC coupled to a QExactive Plus mass spectrometer (Thermo Fisher Scientific). Eluent A was aqueous formic acid (0.1%) and eluent B was formic acid (0.1%) in 80% acetonitrile. Samples were injected directly into a trapping column (PEPMAP NEO C18, 5 μm particle size 300 μm × 5 mm) and separated in the analytical column (EASY-Spray C18 PepMap, 100 Å, 150 mm × 75 μm ID and 3 μm particle size) at a flow rate of 0.25 μL/min. Column temperature was set at 35 °C. Permethylated glycan separation occurred using a multistep linear gradient to obtain 12% eluent B at 10 min, 45% eluent B at 20 min, 60% eluent B at 55 min and 99% eluent B at 65 min. The column was maintained at 99% eluent B for 10 min before re-equilibration at 5% eluent B. The mass spectrometer was operated in the positive ion mode, with an *m/z* range from 500 to 4000 with 140k resolution (Full MS), a spray voltage of 1.9 kV, and a transfer capillary temperature of 275 °C. Tandem MS (MS/MS) data were acquired using a data-dependent method with dynamic exclusion of 5.0 s at a 17,500 resolution. The top 15 most intense ions were selected for higher energy collisional dissociation (HCD), using 10% normalized collision energy (nce), and an isolation window of 4.0 *m/z*. Data were recorded with Thermo Scientific Xcalibur software version 4.5. Instrument performance during nanoLC-MS/MS was monitored using repeated injections of bovine serum albumin (BSA) tryptic digests to ensure consistency. Glycan identifications were validated through MS/MS fragmentation patterns, and specificity was confirmed using PNGase F-treated control samples. To further assess analytical reproducibility, selected samples were reinjected and reanalyzed after three months of dry storage at -20 °C in sealed tubes, yielding consistent glycan profiles with a coefficient of variation (CV) below 10%, confirming method stability. Glycan structures were identified considering previous knowledge on glycosylation, chromatography retention times, and *m/z* identification, assisted by the GlycoWorkbench version 2.1 49. The relative abundance resulted from the sum of the extracted ion chromatogram areas for each glycan structure in relation to the sum of chromatographic areas of all identified glycans.

### Glycoproteomics

GC samples analyzed by *N*-glycomics (12 out of 13) were submitted to lectin-based enrichment to identify putative carriers of paucimannosidic *N*-glycans. Briefly, samples were deparaffinized with heptane and rehydrated, followed by protein extraction using Qproteome FFPE tissue kit (Qiagen), according with manufacture instructions. Total protein extracts were resuspended in GNL buffer (10 mM HEPES, pH 7.5, 0.15 M NaCl, 0.1 mM CaCl_2_) and 200 μg were loaded on agarose-bound GNL for enrichment (Vector Laboratories). After washing the column 10 times with GNL buffer, the glycoproteins were eluted with 3% acetic acid (100 μL for 3 times) and dried in speedvac. Afterwards, samples were resuspended in 50 mM ammonium bicarbonate and incubated at 80 °C for 10 min Glycoproteins were subsequently reduced with 5 mM DTT (Sigma-Aldrich) in 50 mM ammonium bicarbonate at 60 °C for 30 min, followed by alkylation with 10 mM iodoacetamide (Sigma-Aldrich) in 50 mM ammonium bicarbonate for 30 min in the dark. Proteins were then digested with trypsin (Promega) overnight at 37 °C in a humidified chamber. The digestion was quenched by the addition of trifluoroacetic acid (TFA), and the samples were dried using a speed vacuum concentrator. Finally, the dried samples were resuspended in 2% acetonitrile and 0.2% formic acid before analysis by mass spectrometry. The mass spectrometry analysis was performed by nanoLC-MS/MS using a Vanquish neoUHPLC nano-LC coupled to a QExactive Plus mass spectrometer (Thermo Fisher Scientific). Glycopeptides were separated in the analytical column (EASY-Spray C18 PepMap, 100 Å, 150 mm × 75μm ID and 3 μm particle size) at a flow rate of 0.25 μL/min, using a linear gradient of 12-46% eluent B over 50 min. Column wash and re-equilibration were warranted before the following injection. Column temperature was set at 35 °C. The mass spectrometer was operated in the positive ion mode, with an *m*/*z* range from 300 to 2000, a spray voltage of 1.9 kV, and a transfer capillary temperature of 275 °C. Q-Exactive Plus settings were full scan resolution 140k, automatic gain control (AGC) of 3e6, maximum injection time of 200 ms. The top 15 peaks were selected for HCD fragmentation, using the following settings: fragment scan resolution 17,500, fragment scan fixed first mass at 110 *m/z*, AGC target of 1e5, maximum injection time 100 ms, and isolation window 4.0 *m/z*. Data-dependent parameters were as follows: minimum AGC target 7e3, exclusion of charge unassigned, 1, and > 8, peptide match preferred, exclude isotopes on, and dynamic exclusion of 30 s. Two MS runs were performed differing on nce applied: nce of 20% and stepped nce of 35 with 2 steps. Although technical replicates were not feasible due to limited FFPE material, instrument performance was consistently monitored using BSA tryptic digests throughout the LC-MS/MS runs. Mass spectrometry data was processed using the SequestHT search engine and the Percolator algorithm (Proteome Discoverer 3.0, Thermo Fisher Scientific) to validate protein identifications. Data were searched against the human proteome from the SwissProt database (accessed on November 16^th^, 2021). Trypsin was selected as the digestion enzyme, considering up to 3 missed cleavage sites, a precursor ion mass tolerance of 10 ppm, and a product ion tolerance of 0.02 Da. Fixed and variable modifications included carbamidomethylcysteine (+57.021 Da) and oxidation of methionine (+15.995 Da), respectively. The following *N*-glycans were also considered as variable modifications on asparagine (Asn-X-Ser/Thr; “X” does not correspond to Pro), building on glycome analysis: H_2_N_2_F_1_ (+876.322 Da); H_2_N_2_ (+892.317 Da); H_3_N_2_F_1_ (+1038.375 Da); H_5_N_2_ (+1216.423 Da); H_6_N_2_ (+1378.476 Da).

### Glycoprotein annotation and data curation

Identified proteins with high FDR confidence in the Proteome Discoverer analysis were comprehensively curated to identify relevant glycoproteins in GC progression and aggressiveness. Firstly, the generated glycoproteomic dataset was interrogated in terms of cellular location using Panther software (GO terms GO:0005886, GO:0005737, GO:0005634 and GO:0005840) (https://www.pantherdb.org) 50. Subsequently, unique glycoproteomics signatures in the different analysis groups (early-stage versus advanced-stage tumours) were identified using a Venn Diagram analysis (https://bioinformatics.psb.ugent.be/webtools/Venn/). Furthermore, glycoproteins identified across groups were analyzed in terms of differential expression levels using Volcano Plots. An absolute Log2 fold change > 1 and an adjusted *p*-value < 0.05 (-Log10 p-value > 1.3) were considered in the Volcano Plot analysis. Unique and overexpressed glycosignatures in each group were analyzed in terms of their biological and molecular functions using ClueGO plugin from Cytoscape (http://www.cytoscape.org/), considering only significant pathways (*p*-value < 0.05) 51, 52. Putative *N*-glycosylation sites on the identified carriers of paucimannosidic *N*-glycans were evaluated using Net*N*Glyc version 1.0 (https://services.healthtech.dtu.dk/services/NetNGlyc-1.0) 53.

### Glycogene transcript analysis in cancer tissues

Total RNA from 11 FFPE GC tissues characterized by *N*-glycomics was isolated using High Pure FFPET RNA Isolation Kit (Roche) and converted into cDNA with the High Capacity cDNA Reverse Transcription Kit (Applied Biosystems). *HEXA, MAN2A1* and *MGAT2* gene expression was assessed by quantitative polymerase chain reaction (qPCR) and mRNA levels were normalized to the expression of *GAPDH* (glyceraldehyde-3-phosphate dehydrogenase). The relative mRNA levels were calculated using the formula 2-ΔΔCt as described by Livak et al. 54. All reactions were run in duplicates.

### Transcripts and glycoprotein expressions* in silico*

mRNA expression levels of main glycosidases and glycosyltransferases *(HEXA*, *HEXB*, *MAN2A1*, *MAN2A2*, *MAN2B1*, *MGAT1*, and *MGAT2*) involved in paucimannose biosynthesis as well as *MMP9* were examined within a dataset comprising 412 gastric cancer cases. The analysis was conducted by re-examining data from the Genomic Data Commons (GDC) repositories (TCGA-STAD dataset) 55. Detailed clinicopathological data on these data is presented in **Table 5**. Main outcomes included associations with stage, grade, metastasis and prognosis. In addition, we also compared the expression levels of these genes across healthy gastric mucosa, gastric cancer tissue, and histologically normal mucosa adjacent to tumors based on data sourced from The International Cancer Genome Project's Pan-Cancer Analysis of Whole Genomes (PCAWG) available at the EMBL-EBI repository (accession E-MTAB-5200) 56. In addition, glycoproteins linked to advanced stage disease and identified by MS/MS-based GNL-enrichment were assed in 31 healthy human tissues based on proteomics data from the EMBL-EBI repository (accession PXD010154) 57. MMP9 expression levels in healthy tissues was also assessed through the generated antibody-based data from the Human Protein Atlas database (proteinatlas.org) 58.

### Immunohistochemistry for GNL, established GC biomarkers and MMP9

Both gastric tumours of different stages and histologically normal gastric mucosa were screened for GNL staining, established GC biomarkers and MMP9 expressions. Briefly, 3 μm tissue sections were deparaffinized, rehydrated and incubated for 20 min with boiling citrate buffer (Vector Laboratories) or EDTA pH 9. Tissue sections were then exposed to 3-4 % hydrogen peroxide for 5 min (Leica). GNL-ligands were detected using biotinylated GNL (B-1245-2; Vector Laboratories), E-cadherin using mouse anti-human E-cadherin (NCL-L-E-Cad; Leica), p53 using mouse anti-human p53 (M7001, Dako), MSI using antibodies anti-MLH1 (550838; BD Biosciences), anti-MSH2 (556349; BD Biosciences), anti-MSH6 (610918; BD Biosciences), anti-PMS2 (NCL-L_PMS2; Leica), sTn using mouse anti-tag-72 (ab199002; Abcam), sialyl-Lewis A using mouse anti-human CA19.9 (ab116024; Abcam), and MMP9 using rabbit anti-MMP9 (ab76003; Abcam). The Vectastain ABC kit (Vector Laboratories) and the Novolink Max Polymer DS Kit (Leica) were used, according to the manufacturer instructions, to detect biotinylated GNL and primary antibodies, respectively. Positive and negative controls were run in parallel for these antigens to validate the specificity and reliability of the staining process. Controls were elected based on the annotated expression in healthy tissues from The Human Protein Atlas database (proteinatlas.org) 58. For GNL binding, negative controls also included PNGase F treated tissue sections (for *N*-de-glycosylation using 15 U of PNGase F per slide). Enzymatic controls using α-neuraminidase from *Clostridium perfringens* (Sigma-Aldrich) were applied to verify the specificity and accuracy of the immunohistochemical results for the two sialoglycans, sLeA and sTn. Semi-quantitative analysis of GNL, MMP9, E-cadherin, sTn and sLeA was performed, evaluating the percentage of positively stained cells (extension) and the intensity of the chromogenic signal. The final score for each marker was calculated as the product of extension x intensity, providing a composite measure of expression. The MSI phenotype was defined by the loss of expression of any of the analyzed mismatch repair proteins. Conversely, intense nuclear accumulation of p53, as detected by immunohistochemistry using the DO-7 antibody, was interpreted as indicative of underlying *TP53* gene mutations. Sections were evaluated blindly by two independent observers and subsequently confirmed by an experienced pathologist. Image data was acquired using a BA310 Trinocular microscope with an integrated MOTICAM X5 Plus and a Motic ImagePlus 3.0 software (Motic).

### Immunofluorescence

A selection of FFPE tissue sections positive for MMP9 and/or GNL were screened for both antigens through double immunofluorescence to determine colocalization between both epitopes. Tumor and healthy gastric mucosa sections that were positive for only one of these markers or negative for both were screened in parallel as controls. Briefly, FFPE tissues were deparaffined, hydrated and exposed to antigen retrieval with EDTA 1 mM pH8. GNL-ligands and MMP9 were detected using biotinylated GNL (B-1245-2; Vector Laboratories) and anti-MMP9 (ab76003; Abcam), respectively. An anti-rabbit Alexa Fluor 594 (Thermo Fisher Scientific) was used to detect anti-MMP9 primary antibody, and a streptavidin-Alexa Fluor 488 was used as secondary fluorescent detector for the biotinylated GNL. Nuclear counterstain was reached using 4',6-Diamidino-2-phenylindole dihydrochloride (DAPI, Thermo Fisher Scientific). Fluorescence images were acquired on Biotek Cytation C10 Confocal Imaging Reader (Agilent) and generated imaging data was processed and analyzed using Fiji software package. Co-localization analysis was performed by generating a co-localization pixel map through the Colocalization Threshold Test in Fiji software, providing insights into the spatial relationship between the detected antigens.

### MMP9 immunoprecipitation

MMP9 protein was isolated from total proteins extracts excised from MMP9-GNL positive tumour tissues (300 μg) through immunoprecipitation using 3 μg of anti-MMP9 antibody (ab76003; Abcam) adsorbed to the surface of Pierce ^TM^ Protein G agarose beads (Thermo Fisher Scientific), as previously described by us 8. Briefly, after blockage of the Protein G agarose beads with 1 % of bovine serum albumin (BSA, Sigma-Aldrich), protein extracts were precleaned using the blocked agarose beads to reduce unspecific binding. Then, the cleared supernatant was incubated with 3 μg of anti-MMP9 antibody for 2 h at 4 ºC, followed by overnight incubation with BSA-blocked agarose beads. After washing, immunoprecipitated MMP9 was eluted with 3 % acetic acid, dried under vacuum and digested with trypsin as described above. Generated peptides were analyzed by mass spectrometry under the same conditions of the glycoproteomics analysis.

### GNL and MMP9 profiling in serum

GNL and MMP9 serum levels were assessed in a sample set of 89 individuals, comprising both healthy donors and cancer patients, using dot blotting. Demographic and clinicopathological characteristics are summarized in **Table 6**. Briefly, 50 µg of serum proteins, diluted in PBS, were applied onto a nitrocellulose membrane using the Hybrid-dot Manifold system (1050MM, Bethesda Research Laboratories). Membranes were then blocked with either 50% Carbo-free 1× solution for GNL detection or 3% non-fat milk for MMP9 detection. For GNL analysis, membranes were incubated with biotinylated GNL (1:40000, B-1245-2; Vector Laboratories) for 1 h at RT, followed by detection using the ABC Peroxidase kit (Vector Laboratories). For MMP9, membranes were incubated with primary antibody (1:1000; ab76003; Abcam) for 1 h at RT and detected with an HRP-conjugated anti-rabbit secondary antibody (Thermo Fisher Scientific). Antibody-reactive signals were visualized via chemiluminescence. Positive and negative controls - including BSA and PNGase F-treated samples (to remove *N*-glycans) - were included in parallel to validate specificity.

### Statistical analysis

Parametric and non-parametric statistical tests, including t-tests and the Kruskal-Wallis' test, were employed to compare mRNA and protein expression levels across two or more independent groups. Outliers were identified and removed using the ROUT method (Q = 1%) prior to statistical testing, and data normality was assessed using the Shapiro-Wilk test. The mRNA and protein expression levels were analyzed in relation to clinically relevant parameters, such as clinical stage and TNM classification. Chi-square test was used to evaluate the statistical relationships between MMP9-GNL expression and clinical stage, T stage and metastasis. Correlation analyses were performed using Pearson's correlation method for the glycosyltransferases, glycosidases and *MMP9* mRNA levels, while Spearman's correlation was used to assess the correlation between *HEXA, MAN2A1* and *MGAT2* mRNA and paucimannose levels, as between GNL and MMP9 serum levels. The prognosis value of analysed variables was assessed through univariate, and subsequent multivariate Cox regression (co-variables: clinical stage, tumour stage, lymph node involvement, metastasis status). Kaplan-Meier analysis, combined with the log-rank test, was used to compare overall survival curves, with overall survival defined as the time from surgery to the date of patient death. For TCGA data, optimal cut-off value for each gene expression was determined using the *surv_cutpoint* function of the R-package *survminer*, for overall survival outcomes. The clinical value of serum GNL and MMP9 levels was evaluated using Receiver Operating Characteristic (ROC) curve analysis, with the optimal cutoff values determined by the Youden Index. A significance threshold of 95% for the null hypothesis was applied. Statistical analysis was conducted using GraphPad Prism 10 (Dotmatics), IBM Statistical Package for Social Sciences (SPSS; IBM) and R (version 4.4.2) for MacOs.

### Data accessibility

The mass spectrometry data have been deposited to the ProteomeXchange Consortium via the PRIDE partner repository with the dataset identifier PXD060153 59. Glycomics data have been deposited at GlycoPost (accession number GPST000546) 60.

## Results

This study addresses the intestinal-type tumors, which account for approximately 42% of all gastric cancer cases in European countries 61. Tumors were classified into two groups based on prognosis: early-stage, confined to the mucosa or submucosa (T1a, T1b), which are more amenable to curative treatment; and advanced-stage (≥ T2), characterized by deeper invasion and often accompanied by lymph node or distant metastases, leading to poorer outcomes (**Tables 1 and 2**; **Figure S1**). The primary objective was to uncover *N*-glycomic alterations associated with disease aggressiveness and to guide subsequent glycoproteome analysis, aiming to identify molecular signatures that could enhance patient stratification and enable precise tumor targeting.

### Advanced stage GC is enriched for trimmed *N*-glycans

**Figure 1:**
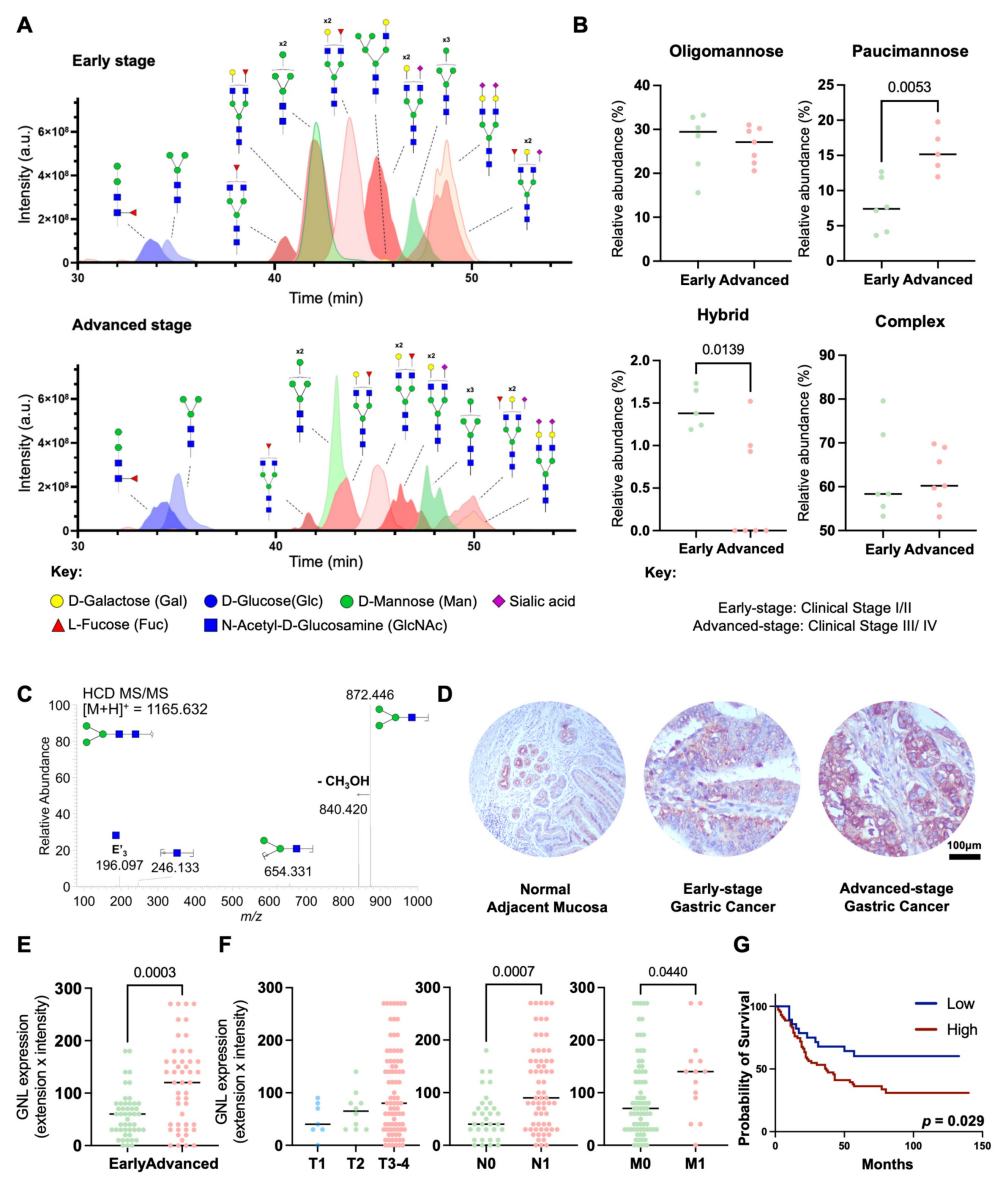
** Advanced-stage GC tumours exhibit increased levels of paucimannosidic *N*-glycans associated with worst prognosis. A. Advanced stage GC (III/IV) exhibit distinctive glycomes compared to early stages (I/II), characterized by higher levels of trimmed *N*-glycans.** The Extracted Ion Chromatograms (EICs) show most abundant *N*-glycans identified in glycomics analysis. Advanced-stage GC tumors exhibit higher levels of specific paucimannoses (blue) and lower levels of complex N-glycans (red) compared to early-stage tumors. In contrast, the relative expression pattern of oligomannoses (green) remains unchanged.** B. Advanced stage tumours expressed higher levels of paucimannoses.** The relative abundance of different classes of *N*-glycans across GC stages (n = 13; stage I/II: 6, stage III/IV: 7) was assessed, revealing lower levels of hybrid *N*-glycans (*p* = 0.0139) and higher levels of paucimannoses (*p* = 0.0053) at advanced stages compared to early stages of the disease. No additional significant associations were observed for the remaining classes of *N*-glycans. Unpaired t-test or Mann-Whitney test were applied, after determination of data normality (Shapiro-Wilk) and outliers' removal. Statistical significance was considered when *p* < 0.05. **C. MS/MS spectrum for the permethylated reduced paucimannose H_3_N_2_ at *m/z* 1165.6.** The MS/MS shows major ions corresponding to the B_3_ fragmentation and the subsequent loss of CH_3_OH, at *m/z* 872.4 and 840.4, respectively. Two internal fragments were also observed at *m/z* 246.1 and 654.3. Additionally, the fragment glycan ion at *m/z* 196.1 results from elimination of the substituents at positions 3 and 4 (E'_3_) in the GlcNAc B-ion. **D-E. GNL staining, used as a surrogate for paucimannose expression, is increased in GC compared to histologically normal mucosa and increases with disease severity.** Immunohistochemical evaluation using GNL lectin revealed the presence of paucimannosidic *N*-glycans in healthy gastric mucosa, particularly in basal glands and intraepithelial immune cells. Advanced-stage tumours exhibited significantly increased expression of these glycans (*p* = 0.0003) predominantly in the cytoplasm of tumour cells but also at the cell membrane. Unpaired t-test was performed, after outliers' removal (ROUT method) and data normality determination (Shapiro-Wilk test). Statistical significance was considered when *p* < 0.05. **F. GNL staining is higher in the primary intestinal-type tumors of metastatic patients**. GNL staining is significantly increased in cases presenting lymph node metastasis (n = 100; *p* = 0.0007) and/or distant metastasis (n = 91; *p* = 0.0421). Kruskal-Wallis's test was used to compare the expression levels in the T stage and Mann-Whitney test was implemented to compare GNL expression in metastatic tumors. Outliers were removed from the analysis and data normality was evaluated using Shapiro-Wilk test. Statistical significance was defined as *p* < 0.05.** G. Patients overexpressing GNL-ligands present the worst prognosis** (*p* = 0.0410). Log-rank (Mantel Cox) was used to compare survival distribution between groups. A *p*-value of less than 0.05 was considered statistically significant.

To address the poorly understood GC *N*-glycome, we extracted *N*-glycans from tumor tissue sections via on-site *N*-deglycosylation using PNGase F. Briefly, *N*-glycans were directly released from minimal amounts of tumor tissue immobilized on glass slides (≥ 10 µm thick), permethylated, and analyzed by nanoLC-MS/MS. A total of 126 distinct glycan structures were identified, with 107 *N*-glycans detected in early-stage gastric cancer and 90 in the advanced-stage group (**Table S1**). The identified *N*-glycans were categorized into four main types: oligomannose glycans, characterized by the presence of 5 to 9 mannose residues; complex glycans, which feature a core of three mannose residues further extended by other sugars; hybrid glycans, combining features of both oligomannose and complex glycans; and paucimannose glycans, which are truncated forms containing up to four mannose residues, often resulting from the enzymatic trimming of recently formed hybrid *N*-glycans (**Figure S2**). Complex *N*-glycans were found as the predominant glycosidic chains in GC (50-80% of total glycans), irrespective of disease stage (**Figures 1A-B; Table S1**). Interestingly, the levels of sialylation, capping glycans often found in these structures and reported to change with cancer progression, remained similar across groups. Other major classes of *N*-glycans included oligomannosidic (15-35%; **Figure 1B**; **Table S1**) and paucimannose structures (5-20%; **Figure 1B, Table S1**), the latter being a relatively underexplored class in humans, thought to arise from the trimming of complex and hybrid *N*-glycans. Hybrid *N*-glycans were also detected in low amounts, comprising less than 2% of the total *N*-glycome (**Figure 1B; Table S1**). Notably, with disease progression we found a significant increase in paucimannose and a decrease in hybrid *N*-glycans (**Figures 1A-B, Table S1**). Also, the main paucimannose structures increased in cancer were H_2_N_2_F_1_ and H_3_N_2_, as highlighted by typical MS/MS spectra (**Figure 1C**).

To further validate and quantify the expression of paucimannose structures, we performed immunohistochemical screening of the tumors using GNL. GNL is specific for α1-6 and α1-3 mannose terminal linkages, which are characteristic of the predominant paucimannose structures identified through mass spectrometry (H_2_N_2_F_1_ and H_3_N_2_; **Table S1**) 62. Notably, although it preferentially binds to paucimannose structures, it also exhibits weaker affinity for hybrid *N*-glycans, which represent a low-abundance population and are reduced in advanced GC (**Figures 1A-B**). Additionally, it binds to oligomannose structures, which remain unchanged during disease progression (**Figures 1A-B**), supporting to be a good approach to assess the expression of trimmed glycans. Accordingly, both healthy gastric mucosa obtained from healthy donors and gastric tumors displayed reactivity to GNL lectin, with staining observed predominantly in the cytoplasm and, to a lesser extent, at the plasma membrane (**Figure 1D**). In gastric tissues, GNL expression was predominantly localized in basal glandular cells and intraepithelial immune cells, being more prevalent in invasive tumour fronts. Beyond the stomach, GNL staining was further observed in cells within the exocrine pancreas, germinal cells of the testis, basal and keratin layers of the skin, and intraepithelial immune cells in the colon (**Figure S3**).

These findings highlight the potential diverse distribution of paucimannose structures across relevant healthy tissues, including epithelial and immune cell populations in both gastric and extra-gastric contexts. Despite these observations, we also found higher GNL reactivity in GC in relation to healthy organs (**Figure 1D**). Furthermore, there was a noticeable increase with the stage and grade of the disease as well as with the presence of lymph node and distant metastases (**Figures 1E-F**). Furthermore, higher GNL reactivity was significantly associated with decreased overall survival (*p* = 0.029; **Figure 1G**), reinforcing the close link between the overexpression of paucimannose and aggressive GC traits. Additionally, half of the metastasized lymph nodes exhibited positivity for GNL (**Figures S4A-C**). Patients with GNL-positive lymph nodes showed decreased overall survival (**Figure S4D**), consistent with observations in primary tumors that link these glycan alterations to increased GC aggressiveness. Notably, based on multivariate analysis, GNL was not observed as an independent predictor of worst prognosis (data not shown). Nevertheless, collectively, our findings link GNL staining to more aggressive cases. We further expanded our patient cohort to include diffuse and mixed-type tumours, which are less prevalent and generally associated with more aggressive clinical behavior in gastric cancer (**Figure S5A-D**). GNL reactivity was significantly higher in intestinal-type tumours compared to diffuse-type, while no significant differences were observed for mixed-type tumours (**Figure S5B**). Notably, although mixed-type tumours were underrepresented in our cohort due to their lower incidence, we also observed elevated GNL reactivity in this subgroup. Additionally, GNL reactivity showed a trend toward association with more aggressive tumour phenotypes and poorer prognosis (*p* = 0.051; **Figure S5D**). Finally, building on reports linking changes in glycosylation to aging 63, 64, we further assessed the effect of age on glycan expression. However, no significant correlation was observed between age and GNL reactivity in our cohort, suggesting that the observed glycomic alterations are more closely related to disease progression rather than age-related physiological changes.

### Prognostic value of GNL vs classical biomarkers

Focusing on intestinal tumours, we assessed the prognostic value of GNL and further compared it with other clinically relevant gastric cancer biomarkers, including E-cadherin, p53, MSI, and the cancer-associated glycans sTn and sLeA. Primarily, we observed significantly decreased E-cadherin expression in advanced-stage tumours, accompanied by a trend toward an association with reduced overall survival (**Figure S6A**). These findings are consistent with previous reports linking E-cadherin loss to tumour progression, poor prognosis, and its role in epithelial-to-mesenchymal transition, a key mechanism driving cancer invasion and metastasis 65-67. On the other hand, accumulation of p53, most likely resulting from underlying mutations, showed no significant association with tumour stage or patient survival (**Figure S6B**) 68. These findings are consistent with report indicating that p53 immunoreactivity alone is not a reliable prognostic marker in GC 69. MSI was determined based on the assessment on the assessment of four DNA repair proteins (MLH1, MSH2, MSH6 and PMS2), following established guidelines for the classification of MSI status. We found higher MSI positivity among early-stage intestinal-type gastric tumours, with significant associations observed with no lymph node involvement, no distant metastasis, and increased overall survival (**Figure S6C**). These findings reinforce previous observations in intestinal-type gastric cancer linking MSS to disease aggressiveness 70. sLeA was associated with distant metastasis, reflecting its known biological role in facilitating cancer cell intravasation into the bloodstream and extravasation at distant sites 71. It was also significantly associated with decreased overall survival (**Figure S6D**). In contrast, the glycan epitope sTn showed no clear association with disease stage or metastatic spread, but was linked to reduced survival, consistent with previous reports (**Figure S6E**) 72, 73. Collectively, these glycan epitopes reflect profound alterations in the tumour glycocalyx and underscore the potential of glycosylation-based biomarkers for patient stratification.

**Figure 2:**
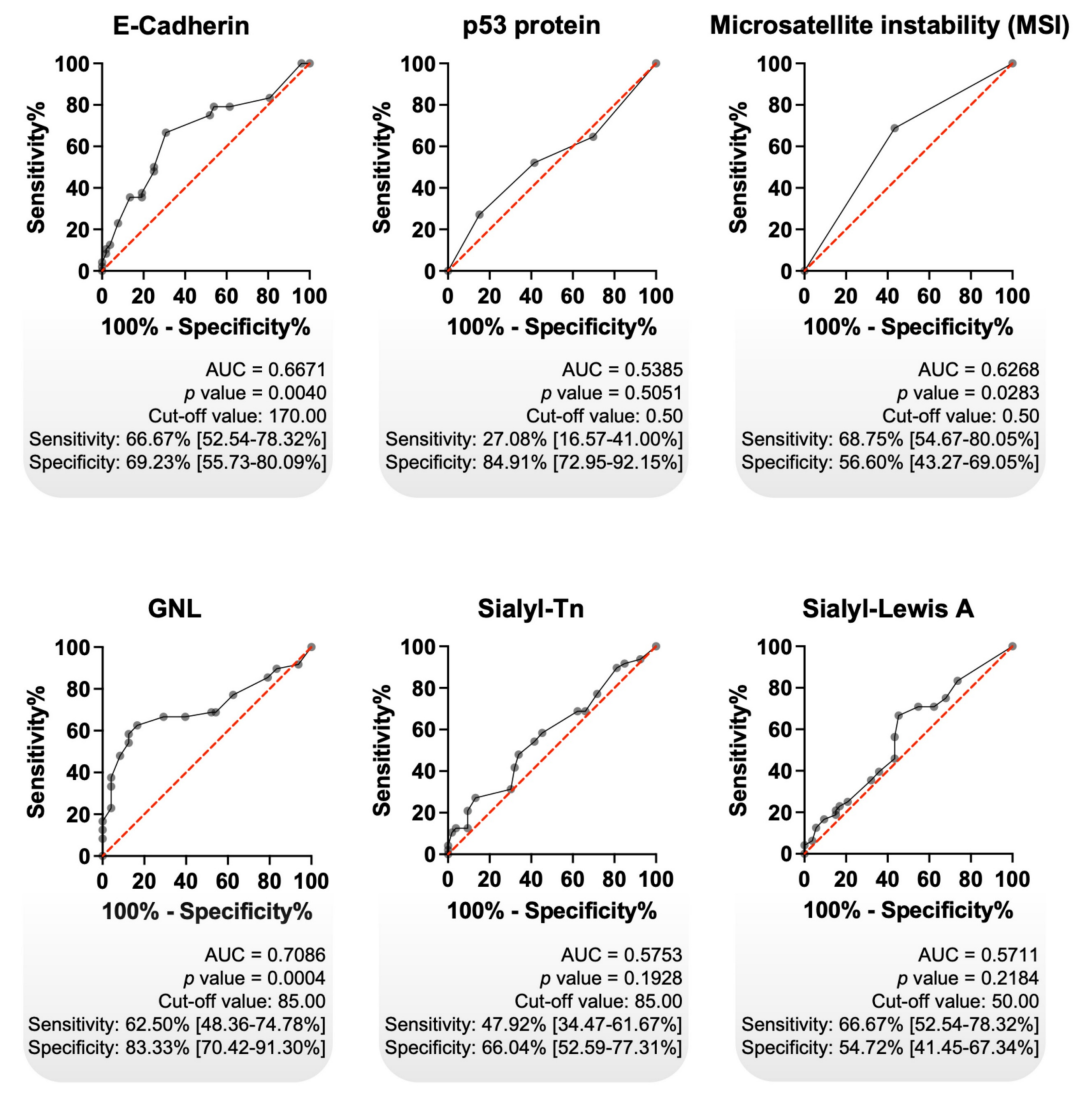
** GNL exhibited the highest diagnostic accuracy among classical GC biomarkers.** Receiver Operating Characteristic (ROC) curve analysis was performed on intestinal-type GC tumours, using immunohistochemistry data, to evaluate the potential of each biomarker in distinguish early-stage from advanced-stage disease (n = 101). GNL showed the highest diagnostic performance (AUC = 0.7086; *p* = 0.0004), with notably high specificity (83.33%), followed by E-Cadherin (AUC = 0.6671; *p* = 0.0040) and MSI phenotype (AUC = 0.6268; *p* = 0.0283). In contrast, p53, Sialyl-Tn (sTn), and Sialyl-Lewis A (sLeA) did not demonstrate statistically significant diagnostic value. The diagonal red line indicates the reference line (random classifier), while the black curves represent the performance of each biomarker. Cut-off values were determined using Youden's index. A *p* value of less than 0.05 was considered statistically significant. AUC stands for Area Under the Curve.

Using receiver operating characteristic (ROC) curve analysis, we compared the prognostic performance of classical biomarkers with GNL, which demonstrated superior predictive capacity (AUC = 0.7086, *p* = 0.0004; sensitivity: 62.5%; specificity: 83.3%; **Figure 2**). Among all tested biomarkers, GNL yielded the highest AUC, as well as the best balance between sensitivity and specificity (**Figure 2**). Additional analyses combining GNL with other biomarkers did not result in a relevant improvement in predictive performance (data not shown). These results indicate that GNL possesses clinically relevant prognostic potential in intestinal-type gastric cancer, outperforming classical biomarkers in both statistical significance and overall discriminative ability. Its high specificity is particularly promising for stratifying patients at risk of poor outcomes, although the moderate sensitivity suggests it may benefit from combination with complementary markers in future models. Nonetheless, GNL stands out as a strong candidate for further validation as a prognostic biomarker.

In an effort to explore the potential of GNL as a non-invasive circulating biomarker, we also evaluated its detection in serum samples from a subset of the patient cohort (**Table 6**). However, GNL levels in serum did not distinguish cancer patients from individuals with non-malignant pathologies (**Figures S7A-C**). Furthermore, it did not show any correlation with age (data not shown). Moreover, it showed no significant association with histological subtypes (intestinal, diffuse, mixed), disease stage, metastatic status, or overall survival (**Figures S7D-F**). These findings suggest that while GNL exhibits strong prognostic value in tissue, its utility in serum may be limited, potentially due to low circulating abundance, interference from abundant serum glycoproteins, or altered glycan presentation. Further methodological optimization and assay refinement will be necessary to evaluate its potential for liquid biopsy applications.

### Glycogenes linked to paucimannose biosynthesis

**Figure 3:**
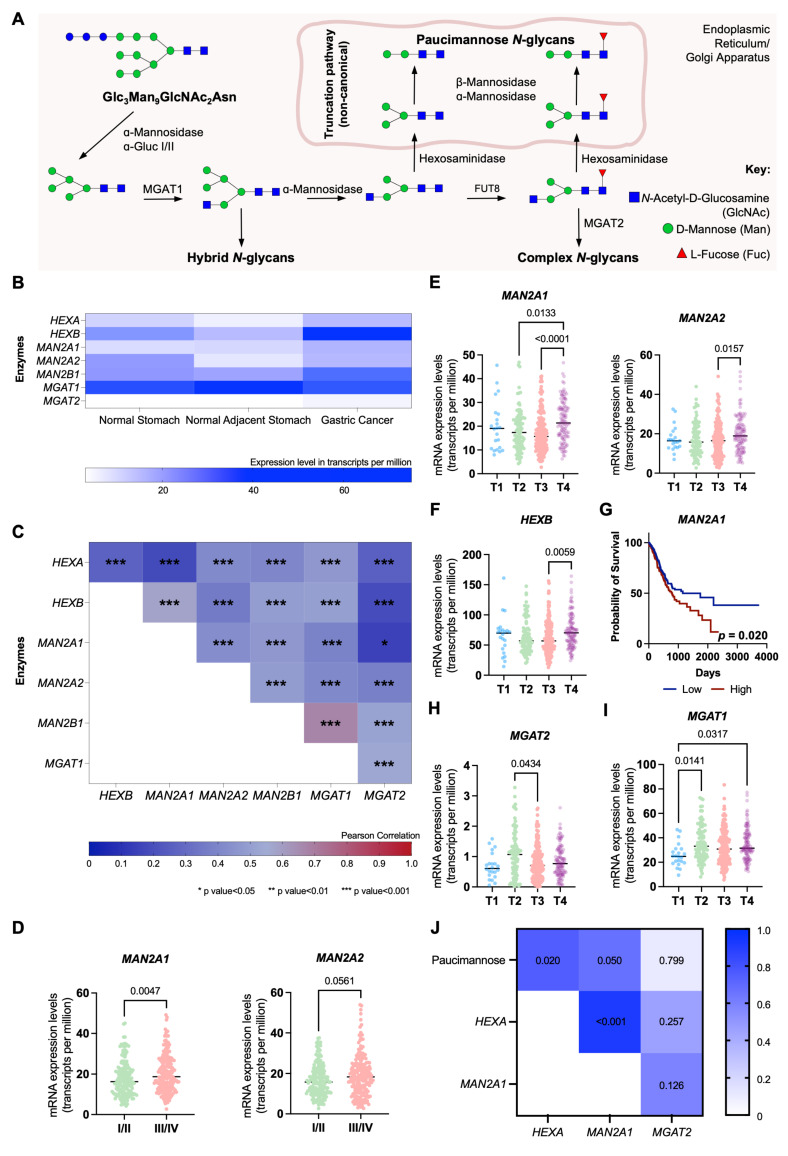
** Aggressive forms of GC significantly upregulate key glycosidases linked to paucimannose expression. A. *N*-glycan biosynthesis pathway highlighting glycosidases and glycosyltransferases regulating glycosidic chain trimming.** α-mannosidases (MAN2A1, MAN2A2), β-mannosidases (MAN2B1) and hexosaminidases (HEXA and HEXB) are the main responsible for trimming *N*-glycans and originating paucimannose. On the other hand, MGAT1 and MGAT2 are amongst the main responsible for the elongation of *N*-glycans. **B. Paucimannose-related glycosidases are significantly overexpressed in GC tissues compared to adjacent healthy mucosa and mucosa from non-GC patients. In contrast, *MGAT1* is predominantly expressed in healthy tissues, and *MGAT2* is minimally expressed. This indicates a microenvironment favoring trimmed *N*-glycan formation in GC tissues. C. Glycogenes associated with *N*-glycan trimming display varying positive correlations (0.150 < R < 0.670, *p* < 0.05) in TCGA GC data (n = 412).** Pearson's correlation was applied to show the level of correlation between the expression of the different glycogenes. **D. Mannosidases (*MAN2A1*, *MAN2A2*) show significant upregulation in advanced-stage (III/IV) compared to early-stage (I/II) GC. E-F. Advanced T-stage tumors (T4) exhibit upregulation of *MAN2A1*, *MAN2A2* (E) and *HEXB* (F) compared to earlier T stages.** *MAN2A1* is significantly upregulated in T4 compared to T2 (*p* = 0.0133) and T3 (*p* < 0.0001), while *MAN2A2* is elevated in T4 relative to T3 (*p* = 0.0157). *HEXB* is also significantly upregulated in T4 tumors (*p* = 0.0059). **G. Patients with *MAN2A1* upregulation face worse prognosis (*p* = 0.020), as shown by overall survival analysis (Log-rank test). H. *MGAT2* expression is significantly reduced in T3 (*p* = 0.0434) and shows a downward trend in T4 compared to T2 tumors. I. *MGAT1* is significantly upregulated in aggressive tumors, with higher expression in aggressive tumours (T2: *p* = 0.0141; T4: *p* = 0.0317) compared to T1**. **J. Gastric tumours from IPO-Porto's patient set revealed a significant correlation between glycosidases and paucimannose relative expression according to glycomics.** A positive correlation was observed between the mRNA levels of *HEXA* and *MAN2A1* and the relative abundance of paucimannosidic *N*-glycans in GC tumors (*HEXA*: R = 0.750, *p* = 0.020; *MAN2A1*: R = 0.667, *p* = 0.050). Additionally, the expression levels of *HEXA* and *MAN2A1* were strongly correlated (R = 0.917, *p* < 0.001) as observed for TCGA data (A). Correlations were assessed using Spearman's correlation test. Otherwise stated, data were assessed for normality (Shapiro-Wilk test), and outliers were removed using the ROUT method. Tests used include Mann-Whitney for analysis in panel D and Kruskal-Wallis for panels E-I. For all analyses, statistical significance was set at *p* < 0.05.

We further explored the expression of glycogenes involved in the trimming of hybrid *N*-glycans leading to paucimannose structures, exploiting the Expression Atlas and The Cancer Genome Atlas Program (TCGA) databases comprehending over 400 GC cases of different stages and grades of the disease (**Table 5**). These include genes encoding for multiple glycosidases (*HEXA*, *HEXB*, *MAN2A1*, *MAN2A2*, *MAN2B1*) and glycosyltransferases responsible for mannose core extensions, such as *MGAT1*, linked to initiating the first branch in complex, as well as hybrid, *N*-glycans, and *MGAT2*, which facilitates the formation of bi-antennary structures (**Figure 3A**). All glycosidases and glycosyltransferases, except *MGAT1*, were significantly elevated in GC compared to adjacent normal gastric mucosa (**Figure 3B**). Moreover, *HEXA*, *HEXB*, *MAN2A1*, and *MAN2B1* were overexpressed in cancer relative to healthy stomachs from individuals without gastric pathologies (**Figure 3B**). Additionally, all glycosidases showed strong positive correlations in gastric tumors (**Figure 3C**), indicating a permissive microenvironment for the accumulation of trimmed glycans in cancer. **Figures 3D-F** highlight clinically significant features, including elevated *MAN2A1* and *MAN2A2* expressions in advanced clinical stages (III/IV) and T stages (T2-T4), and *HEXB* specifically in T4 tumors (**Figures 3D-F**). Additionally, *MAN2A1* overexpression was significantly associated with reduced survival (*p* = 0.020; **Figure 3G**), while *HEXA*, despite not increasing with disease progression, showed a trend toward reduced survival (*p* = 0.097; **Figures S8A-B**). In addition, we also observed a statistically significant decrease in *MGAT2* expression in T3 tumors (**Figure 3H**). These findings highlight a link between paucimannose-related glycogenes and cancer aggressiveness, consistent with the glycome analysis (**Figure 1**). Notably, *MGAT1* mRNA levels also significantly increased in advanced T stages (≥ T2), while *MAGT2* showed upregulation in T2 tumors (**Figures 3H-I**). This aligns with the predominance of complex glycans in GC and suggests their co-expression with paucimannoses (**Figures 1A-B**). Furthermore, this suggests that the accumulation of trimmed *N*-glycans in GC tissues may be primarily driven by glycosidase overexpression rather than a downregulation of the glycosyltransferase responsible for mannose branching towards complex glycans. Reinforcing this hypothesis, our data highlight a significant positive correlation between paucimannose levels (**Figures 1A-B**) and the expressions of *HEXA* and *MAN2A1* in tumors (R = 0.67-0.75; **Figure 3J**).

### Proteomics and Glycoproteomics identifies signatures of GC aggressiveness

**Figure 4:**
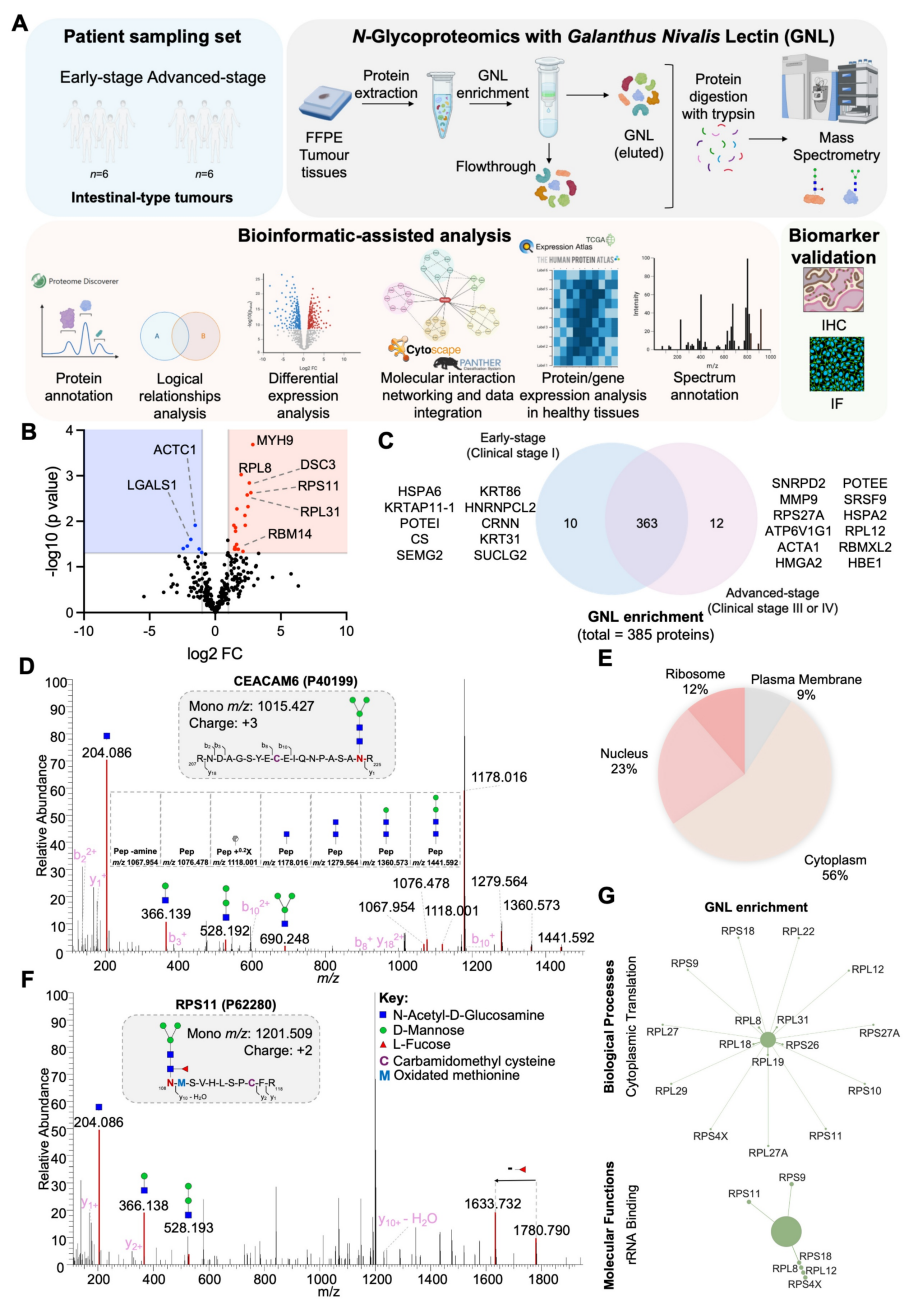
** Paucimannosidic glycoproteome associated with aggressiveness. A. Roadmap for identifying putative carriers of paucimannosidic *N*-glycans.** Proteins were extracted from FFPE GC intestinal-type tumor sections (6 early-stage vs. 6 late-stage tumors) to identify glycoproteome signatures linked to cancer aggressiveness. Glycoproteins potentially carrying paucimannose structures were enriched using GNL-affinity chromatography and identified through conventional bottom-up proteomics using nanoLC-HCD-MS/MS. The presence of *N*-glycans in the identified glycoproteins was confirmed via manual spectral verification and annotation. Relevant paucimannosidic *N*-glycans associated with cancer aggressiveness were validated through various immunoassays. Cartoon elements were created using Biorender.com. **B. GNL-enrichment identified 386 glycoproteins in GC, 18 elevated in advanced stages (red) and 6 proteins in early stages (blue).** Notable proteins in advanced stages include MYH9, RPL8, DSC3, RPS11, RPL31, and RBM14, while ACTC1 and LGALS1 are overexpressed in early stages. **C. GC exhibited unique stage-related GNL-glycoproteomes.** Venn diagram analysis highlights 12 proteins exclusive to advanced stages (e.g., MMP9, RPL12, ACTA1) and 10 proteins unique to early stages (e.g., KRT31, CS, POTEI). **D. CEACAM6, a typical plasma membrane protein, is a carrier of paucimannosidic *N*-glycans.** MS/MS spectrum for the *N*-glycopeptide (_207_RNDAGSYECEIQNPASANR_225_ + H_3_N_2_) highlights the presence of glycan oxonium ions and neutral glycan losses. **E. GNL-enriched glycoproteins were predominantly localized intracellularly.** Glycoproteome data integration based on Gene Ontology (GO) terms for cellular localization, analyzed using the PANTHER bioinformatics tool, reveals an overrepresentation of cytoplasmic proteins (56%). This is followed by lower representations of nuclear (23%), ribosomal (12%), and plasma membrane (9%) proteins. **F. Ribosomal protein RPS11 is a carrier of paucimannosidic *N*-glycans.** MS/MS spectrum support the existence of H_3_N_2_F_1_ *N*-glycan on the peptide _108_NMSVHLSPCFR_118_. The spectrum presents the glycan oxonium ions at *m/z* 244.086, 366.139 and 528.190, and a loss of fucose moiety, supporting the existence of this paucimannosidic *N*-glycopeptide. H, N and F stands for D-Mannose, N-Acetyl-D-Glucosamine and L-Fucose, respectively. **G. Glycoproteins potentially carrying paucimannose structures were predominantly associated with cytoplasmic translation processes in advanced stage tumours.** Cytoscape analysis of unique and overexpressed glycosignatures in advanced-stage tumors revealed significantly enriched pathways linked to cytoplasmic translation and rRNA binding (*p* < 0.05). In contrast, no significant pathways were identified for early-stage tumors.

To establish a rationale for selectively targeting more aggressive gastric tumors, we characterized both the whole proteome and *N*-glycoproteome of tumor regions excised from FFPE tissues. We employed a GNL-enrichment strategy to selectively isolate *N*-glycoproteins potentially carrying trimmed mannoses, utilizing conventional bottom-up glycoproteomics techniques supported by nanoLC-MS/MS analysis with HCD fragmentation (**Figure 4A**). Our analysis identified 1697 proteins from minute amounts of cancer cells, primarily localized to the cytoplasm (57%), nucleus (23%), plasma membrane (15%), and ribosome (5%) (**Figure S9A**). We identified significant differences between the proteomes of early- and advanced-stage tumors, with proteins like DCN and SGTA underexpressed and IFI30 and TACSTD2 overexpressed as the disease progressed (**Figure S9B**). Notably, 18 glycoproteins were exclusive to early-stage tumors, while 8 were found only in advanced stages (**Figure S9C**). Early-stage tumors displayed enrichment in proteins associated with stress adaptation (e.g., HMGB1, HSP90AA2P, MT1M, GSTA1), immune system interactions (HLA-DRB3, C4B, HMGB1), and invasion/migration (SPRR2D, CNFN, RAB13, KRT6C). These findings also suggested metabolic shifts (RAB3C, RAB13, DYNLL1) and cytoskeletal remodeling (MYL1, TUBA3D, KRT6C), supporting enhanced motility and invasiveness. Stress-related proteins further hinted at mechanisms of resistance to drug-based therapy. GO term analysis reinforced these observations, highlighting enrichment for extracellular binding and processes linked to cell adhesion and cytoskeletal remodeling (**Figure S9D**). Interestingly, there was also an enrichment for substantia nigra development, a process critical for midbrain functionality (**Figure S9D**). This suggests tumors might adopt neuronal mimicry traits, acquiring plasticity, forming synapse-like structures, and expressing neuronal markers, potentially aiding in evasion of immune surveillance and enhancing adaptive invasiveness, as previously observed for other digestive tract tumours. Collectively, these strategies point out an integrated survival strategy towards sustained growth and spread at early stages. At advanced stages, we identified elevated levels of proteins associated with key cancer hallmarks, including vesicular trafficking and cellular signaling (RAB3B, RAB5B), immune modulation (HLA-DRB1), stress response and protein folding (COA4, ST13P4), epigenetic regulation (ANP32CP), oxidative stress defense (MT2A), and genomic stability (H2BC1) (**Figure S9C**). These changes support processes critical for tumor progression, such as enhanced survival, immune evasion, chemoresistance, and metastasis. A detailed GO terms enrichment analysis highlighted significantly altered molecular functions, emphasizing disruptions in kinase activities, regulation of protein synthesis and degradation, and extracellular matrix interactions (**Figure S9E**). Moreover, advanced tumors demonstrated enrichment in proteins involved in RNA splicing and antigen processing and presentation, suggesting broad reprogramming of molecular pathways to sustain aggressive tumor behavior and immune escape (**Figure S9E**). Collectively, the proteomics analysis underscores the distinct molecular landscapes of early and advanced stages of gastric cancer. It also highlights the utility of FFPE samples as a valuable resource for GC proteome characterization, offering potential for biomarker discovery and therapeutic insights.

GNL enrichment led to the identification of 385 proteins, with 250 predicted to contain *N*-glycosites (**Table S2**), and 96% detected across both early and advanced stages of the disease. However, the unequivocal confirmation of *N*-glycosites carrying paucimannose structures was achieved for only thirteen glycoproteins, likely due to the low abundance of available material. Among the identified proteins, 6 were more abundant in early stages, while 18 showed increased levels in advanced stages (**Figure 4B**). Interestingly, 10 proteins were exclusively detected in early stages, compared to 12 unique to advanced stages (**Figure 4C**). Notable findings of well-known GC-associated glycoproteins carrying trimmed glycosylation include plasma membrane glycoproteins such as CEACAM6 (**Figure 4D**) and secreted glycoproteins like MMP9. Despite these findings, 91% of the proteins were of intracellular origin, consistent with prior studies linking paucimannosidic *N*-glycans predominantly to intracellular proteins (**Figure 4E**) 17, 74. Examples include advanced-stage-associated proteins such as RPS11, a ribosomal protein confirmed to bear paucimannose residues, also showing a typical MS/MS consistent with the presence of these glycans (**Figure 4F**). In addition, several high-confidence non-glycosylated peptides corresponding to these proteins were identified, further strengthening and validating their assignment in the glycoproteomic analysis (**Figure S10**). Furthermore, advanced-stage-associated proteins carrying paucimannosidic *N*-glycans are linked with cytoplasmic translation and rRNA binding (**Figure 4G**), reinforcing the presence of paucimannose residues in proteins mainly located in the cytoplasm.

### MMP9 carries trimmed mannoses and links to poor prognosis

**Figure 5:**
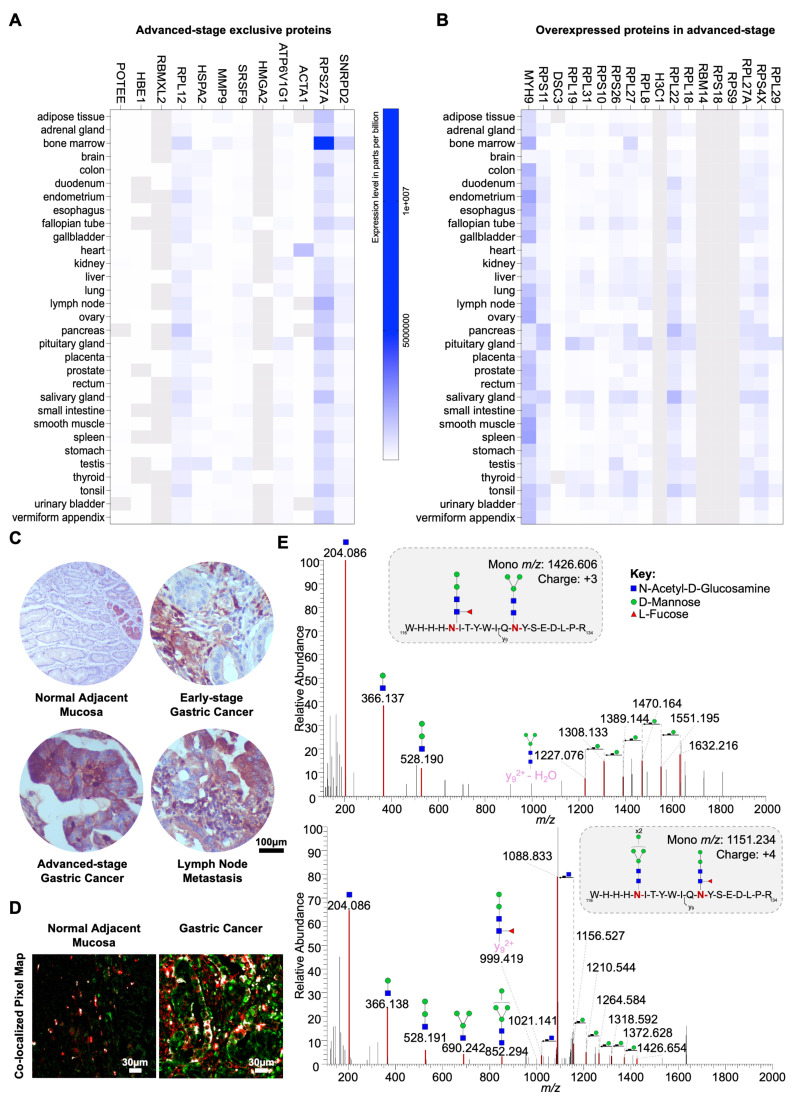
** MMP9 is a key carrier of paucimannosidic *N*-glycans. A-B. *In silico* analysis of human proteome data highlights MMP9 as a highly cancer-specific protein.** An analysis of human tissue proteomics data deposited in EMBL-EBI repository (accession PXD010154) for GNL-enriched glycoproteins overexpressed (**A**) and/or exclusively (**B**) expressed in advanced GC cases highlights the high cancer specificity of MMP9, attributed to its reduced expression in healthy tissues. Residual levels of this protein were detected in the bone marrow, thyroid, and adipose tissue. Proteins with missing data (grey) were excluded from the analysis. **C. MMP9 is overexpressed in advanced-stage tumors and corresponding lymph node metastases compared to healthy tissues and early-stage lesions.** Expression of MMP9 is highly associated with aggressive forms of GC compared to early-stage tumours. Furthermore, it is more expressed in tumours in comparison to the healthy mucosa of non-cancer patients. In tumours, MMP9 was detected on the plasma membrane and cytoplasm of tumor cells, but also in the extracellular matrix (secreted MMP9). In the healthy stomach, its expression is circumscribed to the basal glandular component of gastric epithelium. **D. MMP9 and GNL staining are co-localized in the same tumor areas, suggesting the presence of MMP9 carrying paucimannoses.** MMP9 and GNL staining co-localize in the same tumor areas, suggesting that MMP9 carries paucimannoses. A co-localized pixel map, generated from double immunofluorescence analysis for MMP9 and GNL, revealed co-localized areas (grey/white spots) in tumor sections, in contrast to the normal adjacent mucosa. **E. MS/MS for MMP9 glycoproteoforms carrying paucimannose.** Briefly, MS/MS spectra of MMP9 glycoproteoforms isolated by immunoprecipitation from MMP9-GNL-positive tissues show that it carries H_2_N_2_F_1_ and H_3_N_2_ paucimannosidic residues. MS/MS spectra support the existence of MMP9 peptides carrying paucimannosidic *N*-glycans at the Asn_120_ and Asn_127_ (upper panel) and Asn_127_ (bottom panel). The upper spectrum presents the glycan oxonium ions at *m/z* 244.086, 366.137 and 528.190, and the bottom spectrum presents additional oxonium ions at *m/z* 690.242 and 852.294, according to the identified glycosylation pattern. Moreover, the spectra highlight the presence of peptide fragments carrying H_3_N_2_ (y_9_^2+^ - H_2_O + H_3_N_2_, upper panel) and H_2_N_2_F_1_ (y_9_^2+^ + H_2_N_2_F_1_, bottom panel), reinforcing the presence of these paucimannosidic residues on MMP9. Additional losses of monosaccharide residues (mannose and HexNAc) were identified. H, N, and F represent D-Mannose, N-Acetyl-D-Glucosamine (HexNAc), and L-Fucose, respectively.

We assessed the cancer specificity of proteins elevated or exclusively expressed in advanced-stage patients, enriched via GNL, through an *in silico* analysis of their expression in healthy tissues using human proteome data. MMP9 emerged as the most cancer-specific protein, exhibiting minimal expression in normal tissues, particularly in adipose tissues and immune cells of the bone marrow, pancreas, spleen, and colon (**Figures 5A-B and S11A-B**). In the gastric epithelium, it was mildly expressed in glandular cells and secretions (**Figure 5C**). On the other hand, MMP9 was detected in approximately 55% of the tumors analyzed in the GNL screening, exhibiting strong, widespread expression in the cytoplasm and plasma membrane of cancer cells with a diffuse tumor pattern (**Figure 5C**). It was also highly expressed in tumor-associated immune cells as well as in secretions within the extracellular matrix (**Figure 5C**). Notably, co-expression of MMP9 with GNL staining was observed in overlapping tumor regions in all MMP9 positive cases, encompassing both cellular and secretory compartments (**Figures 5D and S12**). For validation, we immunoprecipitated MMP9 from a tumour excision and analyzed it by mass spectrometry. Tandem mass spectrometry identified characteristic sugar residue fragments, further reinforced the presence of paucimannoses (H_2_N_2_F_1_ and H_3_N_2_) at two of the three known glycosylation sites on MMP9 (Asn_120_ and Asn_127_; **Figures 5E and S13**). We conducted parallel analyses of multiple MMP9 and GNL overlapping areas in five tumors, including the one used for immunoprecipitation assays, as well as corresponding controls and healthy mucosa sections using double staining immunofluorescence. Consistent MMP9-GNL co-expression was observed in these areas, which was not evident in regions positive for only one of these molecules (**Figures 5D and S12**). Furthermore, analysis of healthy gastric mucosa revealed no evidence of abnormal MMP9 *N*-glycosylation (**Figures 5D and S12**), supporting the cancer-specific nature of this glycosylation signature.

**Figure 6:**
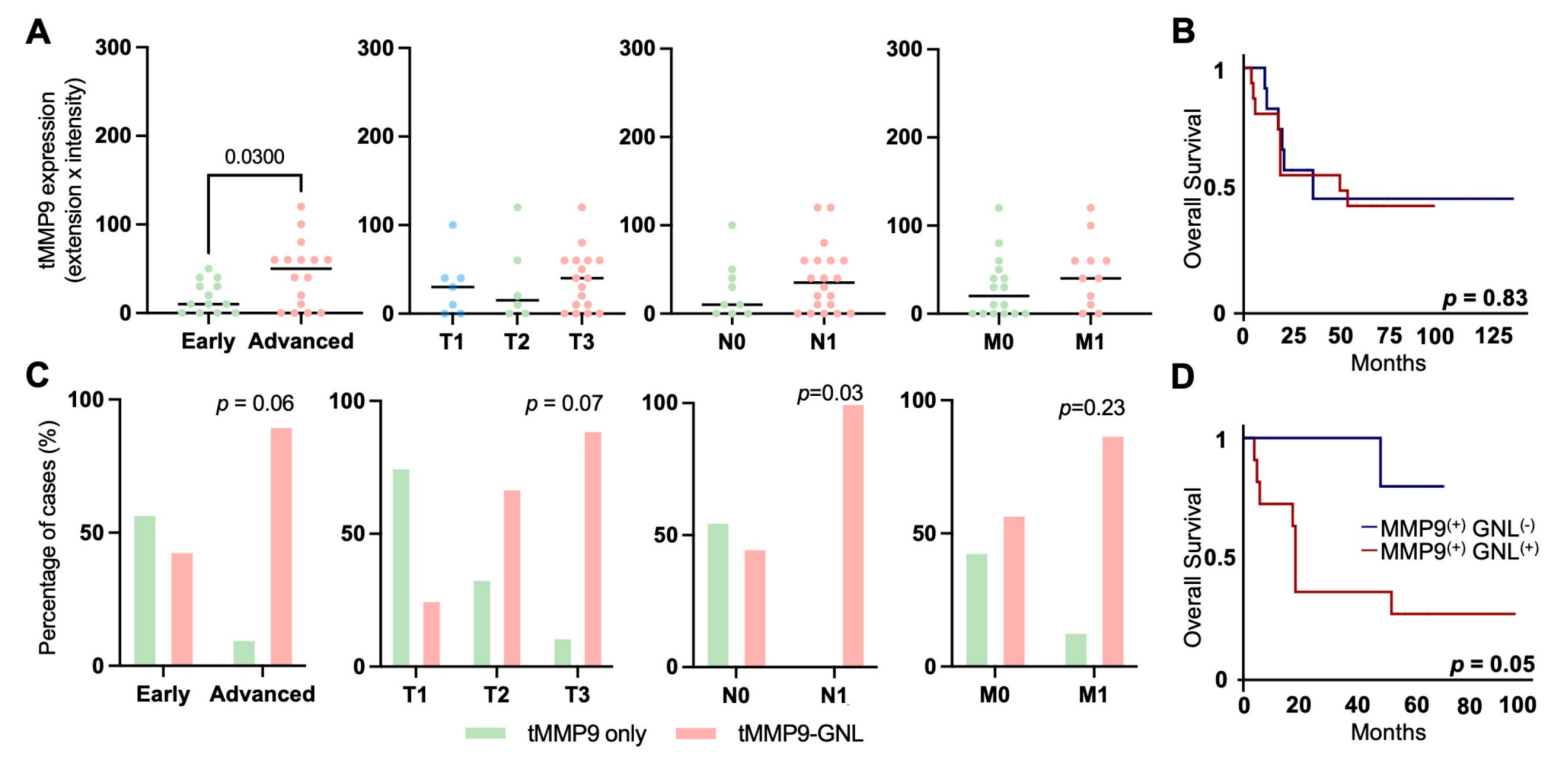
** Abnormally glycosylated MMP9 associates with advanced metastatic tumors and poor prognosis. A. MMP9 expression in tumor cells increases in advanced-stage tumors (n = 30, *p* = 0.0300), with no correlation to TNM classification (*p* > 0.2000).** Statistical analyses included unpaired t-tests for early versus advanced-stage tumors, Mann-Whitney tests for N and M staging, and Brown-Forsythe and Welch ANOVA for mean differences across T stage groups (T1-T3). Outliers were removed using the ROUT method, and data normality was assessed with the Shapiro-Wilk test. Significance was set at *p* < 0.05.** B. MMP9 overexpression does not associate with worse prognosis (*p* = 0.83).** The log-rank test was used to compare overall survival curves, while univariate analysis was applied to assess individual prognostic value.** C. MMP9-GNL positivity shows a trend toward association with cancer aggressiveness, including lymph node metastasis.** The number of MMP9-GNL positive tumors tends to be higher in advanced-stage and high-grade tumors compared to MMP9-GNL negative tumors (early *vs* advanced stage: *p* = 0.06; T3 *vs* T1 and T2: *p* = 0.07). MMP9-GNL-positive cases were significantly higher among tumors presenting lymph node metastasis (*p* = 0.03). The chi-square test was implemented to determine the association between the presence of MMP9-GNL and relevant clinicopathological parameters. **D. Patients presenting the MMP9-GNL phenotype presented unfavorable prognosis compared to MMP9-GNL negative signatures (*p* = 0.05).** The log-rank test was employed to compare overall survival curves, and univariate analysis to evaluate individual prognostic factors.

Clinically, MMP9 levels were significantly higher in advanced-stage compared to early-stage tumors (**Figure 6A**). Notably, MMP9 elevation was not associated with the presence of metastases or worse prognosis (**Figures 6A-B**). Nevertheless, we consistently observed weak to moderate, yet statistically significant correlations (R = 0.2-0.4; **Figure S14**) between *MMP9* expression and all glycogenes involved in *N*-glycan trimming (*HEXA*, *HEXB*, *MAN2A1*, *MAN2A2*, *MAN2B1*). This suggests a degree of co-regulation and aligns with the presence of paucimannose structures in this glycoprotein. To further investigate the clinical relevance of aberrantly glycosylated MMP9, we employed double immunofluorescence assays, combining the detection of MMP9 and GNL lectin staining to assess their co-localization within tumor tissues. While this approach does not conclusively confirm specific *N*-glycan alterations in MMP9, when considered alongside our MS/MS data, it serves as a potential surrogate marker for aberrant glycosylation patterns in this glycoprotein. Based on this premise, we observed higher number of positive cases for MMP9-GNL in advanced-stage compared to early-stage tumors, with a trend toward further increases in T3 and a statistical relation with lymph node metastasis (**Figure 6C**). Furthermore, we found that this phenotype could accurately discriminate patients facing worst prognosis, when compared to assessing MMP9 alone (**Figure 6D**). Nevertheless, based on multivariate analysis, MMP9-GNL positivity was not an independent predictor of worse prognosis (data not shown). In summary, we emphasize the cancer-specific nature of MMP9 and underscore the potential of *N*-glycosylation, particularly the potential acquisition of trimmed glycosignatures, to enhance patient stratification strategies in gastric cancer.

### MMP9 for non-invasive GC detection

**Figure 7:**
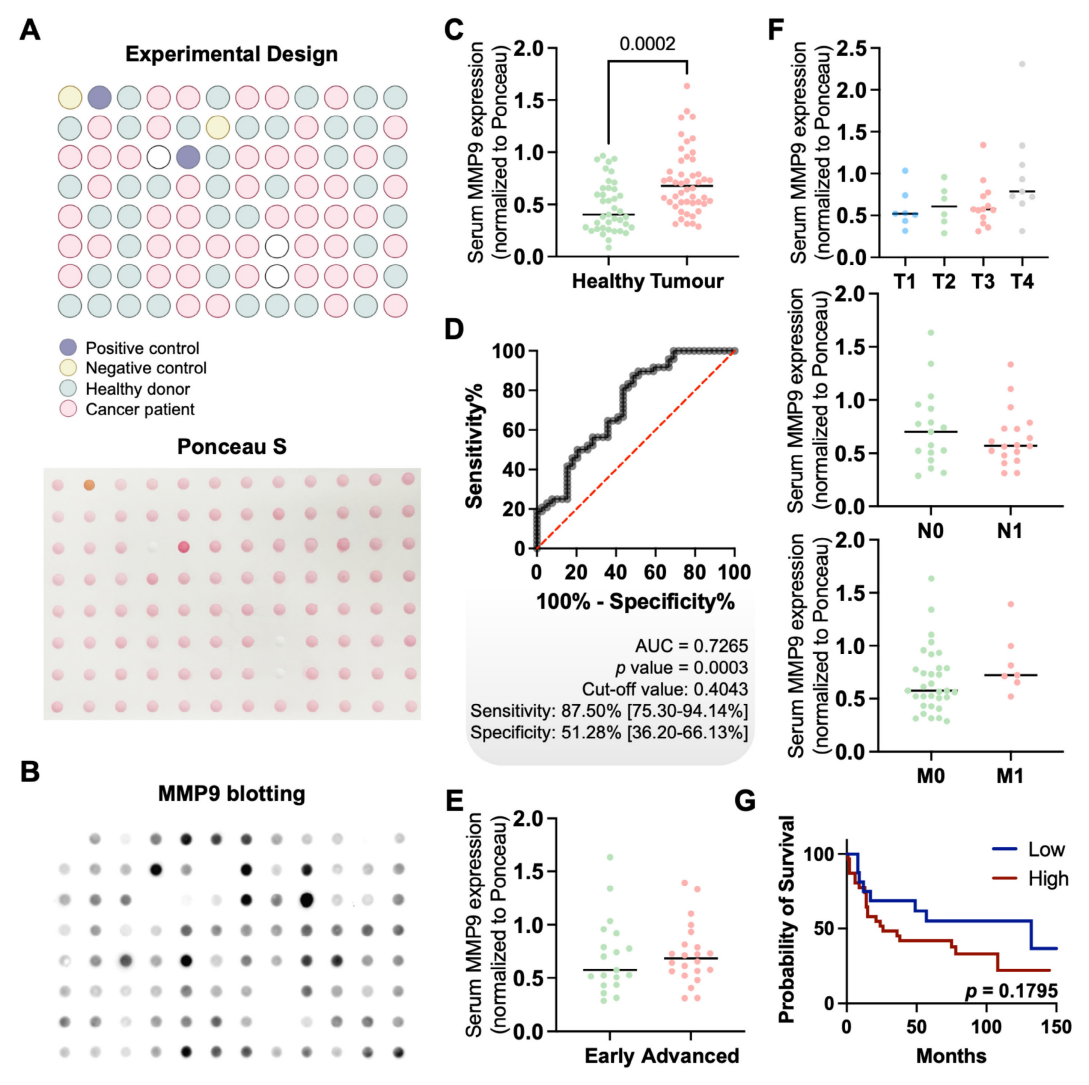
** Serum MMP9 exhibits moderate discriminatory power between healthy individuals and cancer patients. A. Schematic representation of the layout of samples on the Dot Blot membrane.** Dot blot included scattered distribution of cancer patients' samples (pink, n = 49), healthy donors (green, n = 40) and experimental controls (n = 4). Positive and negative controls, comprising proteins extracted from tumour tissue previously evaluated by immunohistochemistry (positive and negative) or bovine serum albumin (negative), were included in experimental design. The Ponceau S staining image confirms uniform protein spotting across all samples. **B. MMP9 dot blotting reveals differential expression levels among samples, with overexpression of MMP9 in cancer patients' serum samples.** Representative illustration of MMP9 immunoblot showing stronger signals in cancer patients compared to healthy donors, validated with accurate positive and negative controls. **C. Cancer patients present increased levels of serological MMP9.** MMP9 was significantly elevated in the serum of cancer patients compared to healthy donors (*p* = 0.0002, Mann-Whitney test). Quantification of MMP9 levels in serum was normalized to Ponceau S. **D. Serological MMP9 exhibits high diagnostic accuracy, distinguishing healthy donors from cancer patients.** The ROC (Receiver Operating Characteristic) curve for serological MMP9 demonstrated a moderate discrimination ability, with an AUC (Area Under the Curve) of 0.7265 (*p* = 0.0003, n = 89), yielding a sensitivity of 87.5% and specificity of 51.28%. The black ROC curve illustrates biomarker performance, with the red diagonal line representing the reference for a random classifier. The optimal cut-off value was determined using Youden's index, and statistical significance was defined as *p* < 0.05. **E and F. Serum MMP9 levels show no significant correlation with the clinicopathological parameters of cancer patients.** No significant correlation was observed between serum MMP9 levels and clinical stage (E) or TNM classification (F), although a trend toward higher MMP9 expression in more advanced disease stages was noted (clinical stage, n = 42; TNM classification, n = 40). A Mann-Whitney test was used to compare serum MMP9 levels between early and advanced-stage tumours. Brown-Forsythe and Welch ANOVA, T test with Welch's correction and Mann-Whitney tests were used to compare the distribution of MMP9 levels across T, N and M stages, respectively. **G. Elevated serological MMP9 levels trend towards reduced overall survival.** Kaplan-Meier survival analysis comparing patients with high versus low serum MMP9 levels showed a trend toward poorer survival in the high MMP9 group, though not statistically significant (n = 49, *p* = 0.1795). Optimal cut-off was defined as the optimal discriminate value in a survival ROC curve. Log rank tests was used to compare overall survival curves. Statistical significance was set at *p* < 0.05. Before performing statistical tests, outliers were removed using the ROUT method (Q = 1%), and data normality was assessed with the Shapiro-Wilk test.

Finally, in an effort to translate these findings into a non-invasive setting, we screened the serum of gastric cancer patients and healthy individuals for MMP9 expression. MMP9 levels were significantly elevated in cancer patients compared to healthy controls (*p* = 0.0002), regardless of histological subtype, stage, or grade (**Figures 7A-F**). Although no statistically significant association with overall survival was observed (*p* = 0.1795; **Figure 7G**), a trend toward poorer outcomes in patients with higher serum MMP9 was noted. ROC curve analysis demonstrated moderate diagnostic performance, with an AUC of 0.7265 (*p* = 0.0003), a sensitivity of 87.5%, and a specificity of 51.28% (**Figure 7D**). These findings highlight the potential of serum MMP9 as a non-invasive biomarker for GC detection. Nevertheless, while its high sensitivity supports the identification of a substantial proportion of cancer cases, the relatively low specificity may limit its standalone diagnostic value, particularly in distinguishing GC from benign or inflammatory conditions that also elevate MMP9 75, 76. In terms of prognostic value, ROC analysis indicated that MMP9 was not suitable for identifying aggressive disease or predicting poor outcomes, irrespectively of the histological subtype (data not shown). This, together with the lack of significant associations with tumor stage or survival, suggests that serum MMP9 may reflect systemic processes rather than tumor-specific aggressiveness. Nonetheless, its robust differential expression between cancer patients and healthy individuals supports its continued investigation, particularly as part of a combined biomarker panel to enhance diagnostic accuracy.

Building on these observations, we further investigated the relationship between serum MMP9 levels and serum GNL expression. No significant correlation was observed between the two biomarkers (**Figure S15**), indicating that they likely capture different aspects of gastric cancer biology. While MMP9 may be more reflective of systemic processes, GNL expression appears to be closely associated with tumour-specific glycosylation patterns, which warrants future investigations. Notably, while GNL has proven effective in detecting a range of mannose-rich structures, its broad binding profile, including hybrid and oligomannose *N*-glycans, may limit its ability to selectively identify specific MMP9 glycoforms of clinical interest. Future studies should aim to define cancer-specific MMP9 glycoproteoforms with higher precision to enhance the specificity of non-invasive detection strategies.

## Discussion

This study significantly advances our understanding of GC glycobiology by revealing novel glycosidic signatures associated with cancer aggressiveness. We observed a significant enrichment of paucimannosidic *N*-glycans in advanced GC, a glycosylation profile not previously linked to this cancer type. While paucimannosylation was once considered limited to invertebrates and plants, its presence in various human tissues, including stem cells, highlights its potential role in cellular development 17, 77. Moreover, its recent association with various pathologies, including several cancers, systemic lupus erythematosus, and inflammatory conditions, indicates a complex interplay between paucimannosylation and disease 18, 21, 77, 78. This study reveals a novel link between paucimannosidic glycans and adverse outcomes in GC, highlighting their potential as biomarkers for disease progression and prognosis. While our findings establish a clear association between paucimannosylation and GC aggressiveness, the functional role of these truncated *N*-glycans in GC remains poorly understood. As such, no direct studies currently address the biological consequences of paucimannosylation in this context. However, reports from other cancer types and inflammatory conditions have shown that paucimannoses can modulate immune responses via interactions with receptors like DC-SIGN and the mannose receptor, and may also impact protein trafficking, receptor stability, and cell signaling 19, 23, 26. Although speculative in the context of GC, similar mechanisms could be envisaged, especially considering the observed enrichment in aggressive tumors. These hypotheses warrant future dedicated functional studies to dissect the biological roles of these glycan structures in GC pathogenesis. Beyond paucimannosylation, a wide spectrum of glycosylation alterations has been documented in GC. Notably, hypersialylation, particularly by overexpression of terminal sLe antigens, has been associated with tumor invasion, immune evasion, and metastasis 29, 30. Complex *N*-glycans with increased branching and terminal sialic acids have also been linked to aggressive disease phenotypes. Likewise, glycan traits such as core fucosylation and bisecting GlcNAc have shown context-dependent roles, sometimes even exerting tumor-suppressive effects 40-43. These diverse and sometimes opposing glycan signatures underscore the intricate regulation of glycosylation in cancer biology. Our findings expand this landscape by highlighting the prognostic relevance of trimmed *N*-glycans, specifically paucimannoses, which remain understudied despite their consistent association with aggressiveness in our GC cohort. Furthermore, we found that the overexpression of multiple glycosidases involved in paucimannose formation in gastric tumours was significantly associated with aggressiveness and paucimannose increase. Specifically, enzymes such as α-mannosidases, which trim high-mannose structures to form paucimannose glycans, showed increased expression in more advanced stages, associating with paucimannose expression. Future studies should elucidate the specific molecular pathways mediating this link and explore the therapeutic and prognostic potential of targeting the glycogenes responsible for *N*-glycan trimming in GC.

Intriguingly, we found that paucimannosidic *N*-glycans are predominantly present in intracellular ribosomal proteins, challenging the traditional view of *N*-glycosylation as a phenomenon limited to the cell surface. This discovery opens new avenues for research into the role of intracellular glycosylation in cancer biology. The presence of these glycans on ribosomal proteins suggests a potential involvement in regulating protein synthesis or ribosome assembly, which could significantly impact cellular processes in cancer cells, warranting further investigation. Nevertheless, we also observe the presence of these glycans on membrane proteins such as CEACAM6 and MMP9, two glycoproteins frequently linked to GC aggressiveness 79-82. Here, we focused on MMP9, a zinc-dependent metalloproteinase that plays a pivotal role in the extracellular matrix 83. MMP9 has been implicated in tumor invasion by degrading the basement membrane, which acts as the first barrier against cancer dissemination across various tumor types 83, 84. Our *in silico* analysis of the human proteome further demonstrated that MMP9 exhibits a restricted expression pattern in healthy tissues, underscoring its potential for precise cancer targeting. In our study, we found that MMP9 levels are significantly elevated in gastric cancer tissues compared to adjacent normal mucosa. Furthermore, increased MMP9 expression correlates with disease stage and grade, and is associated with poor prognosis. These findings align with observations from other studies, underscoring the critical role of MMP9 in the progression and aggressiveness of GC 79, 80. Notably, we also found that MMP9 serves as a major carrier of paucimannoses, supporting that this specific glycosignature could be instrumental in identifying patient subsets with metastasis and worse prognostic outcomes. The paucimannosylation of MMP9 may also have significant implications for its function in GC progression. We speculate that these truncated glycan structures could affect MMP9's enzymatic activity, stability, or interactions with other extracellular matrix components. While further studies are needed to elucidate the functional implications of *N*-glycome remodeling on MMP9 functionality and its role in cancer progression, our findings underscore the importance of this glycoprotein's glycosylation in precise patient stratification and cancer targeting. The association between paucimannosylation and GC aggressiveness also suggests potential targeted therapeutic strategies.

Building on glycomics data, we further used GNL to evaluate paucimannosidic *N*-glycans in GC tissues. This approach was particularly suitable in this context, as oligomannose structures, which are also recognized by GNL, did not show significant variation during disease progression. Accordingly, we observed increased GNL staining associated with intestinal-type gastric cancer aggressiveness and a significant correlation with poorer prognosis. Furthermore, GNL demonstrated superior prognostic performance when compared to classical biomarkers routinely used in clinical practice, including E-cadherin, p53, and MSI. It also outperformed the well-established cancer-associated glycans sLeA and sTn, further reinforcing its association with aggressive tumor behavior in this subtype. Additionally, elevated GNL reactivity in mixed-type tumors suggests its potential for stratifying this challenging and poorly defined subgroup, where reliable biomarkers are limited. These findings support the use of GNL as a promising tissue-based biomarker with potential applicability across multiple histological subtypes. Notably, glycosylation is known to be influenced by various genetic and environmental factors, including smoking, alcohol consumption, and family history 85-88. However, detailed clinical information on these variables was not uniformly available across our cohort, limiting our ability to assess their specific impact in this study. These factors should be addressed more comprehensively in future studies integrating clinical and molecular data to better inform potential clinical translation. Nevertheless, to explore non-invasive clinical applications, we extended our analysis to serum samples. However, GNL showed limited utility in this setting, likely reflecting the challenges of using lectins with broad specificity to detect low-abundance, disease-associated glycans in circulation. This limitation underscores the need for comprehensive serum glycomics analyses in gastric cancer, which remain largely underexplored. In contrast, MMP9 demonstrated moderate diagnostic potential in serum, supporting its value as a candidate for further investigation. Interestingly, we did not observe significant associations with advanced disease features such as stage or lymph node metastasis, as previously reported by other studies 89, 90. Notably, elevated serum MMP9 has also been documented in benign inflammatory conditions, including inflammatory bowel disease, autoimmune disorders such as type 1 diabetes and rheumatoid arthritis, as well as in acute inflammatory settings like ARDS and COVID-19, warranting cautious interpretation in future studies 75, 76, 91. Although we attempted to correlate serum MMP9 levels with GNL reactivity to enhance its predictive value, no significant association was observed. This may reflect the limited specificity of GNL in the serum setting, where it likely fails to selectively detect the glycoforms of interest, in contrast to its performance in tissue-based applications. Nevertheless, our findings from GNL-enrichment-based tissue glycoproteomics suggest that MMP9 may carry disease-relevant glycoforms with potential clinical utility. Therefore, future efforts should focus on characterizing cancer-specific MMP9 glycoforms to enhance specificity and clinical relevance. Targeting such glycoproteoform signatures, rather than relying on general glycan-binding probes, holds strong promise for advancing precision, non-invasive biomarker strategies in GC.

## Concluding Remarks

In summary, these findings reveal promising glycan-based biomarkers for enhanced prognostic stratification in gastric cancer, particularly within the intestinal subtype, which can be detected using GNL. The close association between paucimannosylation, MMP9 expression, and tumor aggressiveness underscores the complexity of glycoproteome remodeling and highlights the potential of glycan-focused approaches to improve patient management. These insights are especially valuable in light of the current lack of robust prognostic tools for histologically defined subgroups. Future research should aim to validate these findings in larger, independent cohorts, investigate the functional impact of *N*-glycan alterations, particularly in relation to MMP9 biology, and explore the therapeutic relevance of glycan signatures, including their potential integration into theranostic strategies.

## Supplementary Material

Supplementary figures.

Supplementary tables.

## Figures and Tables

**Table 1:** Clinicopathological data of patients included in the analysis of GNL and classical biomarkers in GC primary tumors (n = 148).

	*n* (%)	
	
Male	82 (55)	
Female	66 (45)	
	
**Stage**		
I	13 (9)	
II	52 (35)	
III	61 (41)	
IV	22 (15)	
	
**Primary tumor (T)**		
T1	10 (7)	
T2	13 (9)	
T3	87 (59)	
T4	37 (25)	
Missing information	1 (0)	
	
**Regional lymph nodes (N)**		
N0	42 (28)	
N1		32 (22)
N2	20 (14)	
N3	53 (36)	
Missing information	1 (0)	
	
**Distant metastasis (M)**		
M0	115 (77)	
M1	23 (16)	
Missing information	10 (7)	
	
**Lauren's classification**		
Intestinal type	101 (68)	
Diffuse-type	29 (20)	
Mixed	14 (9)	
Missing information	4 (3)	
	
	
**Age** (average, min-max)	64 years (34-89)	
	
	
		

**Table 2:** Clinicopathological data of patients included in the analysis of MMP-9 in GC primary tumors (n = 30).

	*n* (%)	
	
Male	17 (57)	
Female	13 (43)	
	
**Stage**		
I	10 (33)	
II	4 (13)	
III	5 (17)	
IV	11 (37)	
	
**Primary tumor (T)**		
T1	7 (23)	
T2	6 (20)	
T3	16 (54)	
T4	0 (0)	
Missing information	1 (3)	
	
**Regional lymph nodes (N)**		
N0	9 (30)	
N1		8 (27)
N2	3 (10)	
N3	9 (30)	
Missing information	1 (3)	
	
**Distant metastasis (M)**		
M0	16 (53)	
M1	11 (37)	
Missing information	3 (10)	
	
	
**A**ge (average, min-max)	60 years (37-75)	
	
	
		

**Table 3:** Clinicopathological data of patients included in the analysis of GNL-ligands and MMP-9 in GC lymph nodes metastasis (n = 10).

	*n* (%)	
	
Male	5 (50)	
Female	5 (50)	
	
**Stage**		
II	1 (10)	
III	7 (70)	
IV	2 (20)	
	
**Primary tumor (T)**		
T2	1 (10)	
T3	1 (10)	
T4	8 (80)	
	
**Regional lymph nodes (N)**		
N1		1 (10)
N2	0 (0)	
N3	9 (90)	
	
**Distant metastasis (M)**		
M0	7 (70)	
M1	3 (30)	
	
	
**Ag**e (average, min-max)	56 years (39-85)	
	
	
		

**Table 4:** Clinicopathological data of patients included in *N*-glycomics analysis (n = 13).

	*n* (%)
	
Male	10 (77)
Female	3 (23)
	
**Stage**	
I	6 (46)
II	0 (0)
III	5 (39)
IV	2 (15)
	
**Primary tumor (T)**	
T1	4 (31)
T2	3 (23)
T3	5 (39)
T4	0 (0)
Missing information	1 (7)
	
**Regional lymph nodes (N)**	
N0	4 (31)
N1	3 (24)
N2	1 (7)
N3	4 (31)
Missing information	1 (7)
	
**Distant metastasis (M)**	
M0	10 (77)
M1	2 (15)
Missing information	1 (8)
	
Age (average, min-max)	59 years (36-75)

**Table 5:** Clinicopathological characteristics of the GC patients in the TCGA-STAD dataset used in this study (n = 412).

	*n* (%)	
	
Male	267 (65)	
Female	145 (35)	
	
**Stage**		
I	58 (14)	
II	122 (30)	
III	169 (41)	
IV	39 (9)	
Missing information	24 (6)	
	
**Primary tumor (T)**		
T1	22 (5)	
T2	88 (21)	
T3	181 (44)	
T4	113 (28)	
Missing information	8 (2)	
	
**Regional lymph nodes (N)**		
N0	124 (30)	
N1		109 (26)
N2	78 (19)	
N3	82 (20)	
Missing information	19 (5)	
	
**Distant metastasis (M)**		
M0	365 (89)	
M1	26 (6)	
Missing information	21 (5)	
	
	
**Age** (average, min-max)	66 years (30-90)	
	
	
		

**Table 6:** Demographic and clinicopathological profiles of Healthy Donors and GC patients in the GNL and MMP9 serum reactivity analysis (n = 89).

Healthy donors (n=40)	*n* (%)
	
Male	26 (65)
Female	14 (35)
	
Age (average, min-max)	39 years (21-61)
	
Cancer patients (n=49)	*n* (%)
	
Male	20 (41)
Female	29 (59)
	
Stage	
I	11 (23)
II	8 (16)
III	11 (23)
IV	12 (24)
Missing information	7 (14)
	
Primary tumor (T)	
T1	7 (14)
T2	6 (12)
T3	14 (29)
T4	9 (18)
Missing information	13 (27)
	
Regional lymph nodes (N)	
N0	17 (35)
N1	3 (6)
N2	7 (14)
N3	9 (18)
Missing information	13 (27)
	
Distant metastasis (M)	
M0	33 (68)
M1	7 (14)
Missing information	9 (18)
	
Lauren's classification	
Intestinal type	24 (49)
Diffuse-type	10 (20)
Mixed	4 (8)
Missing information	11 (23)
	
	
Age (average, min-max)	63 years (36-83)
	
	

## Abbreviations

AGC: Automatic Gain Control
BSA: Bovine Serum Albumin
DAPI: 4',6-Diamidino-2-phenylindole dihydrochloride
DTT: Dithiothreitol
EICs: Extracted Ion Chromatograms
FFPE: Formalin-Fixed Paraffin-Embedded
GC: Gastric Cancer
GDC: Genomic Data Commons
GNL: Galanthus Nivalis Lectin
GO: Gene Ontology
HCD: Higher energy Collisional Dissociation
NCE: Normalized Collision Energy
PCAWG: International Cancer Genome Project's Pan-Cancer Analysis of Whole Genomes
qPCR: Quantitative Polymerase Chain Reaction
RNA: Ribonucleic Acid
rRNA: Ribosomal Ribonucleic Acid
sTn: sialyl-Tn
TCGA: The Cancer Genome Atlas
TFA: Trifluoroacetic Acid
