# Supplementary material for: Trimmed N-glycans define aggressive gastric cancer and predict clinical outcomes
Source: Theranostics. 2025 Aug 22;15(18):9375–98. doi: 10.7150/thno.111670 (PMC12486017; doi:10.7150/thno.111670)
Supplement: Supplementary file 1 — Supplementary figures. [file thnov15p9375s1.pdf]

Trimmed *N*-glycans define aggressive gastric cancer and predict clinical outcomes

Supporting information

Dylan Ferreira[1,2,3], Beatriz Marinho-Santos[1,2], Marta Relvas-Santos[1,2], Bernardo Orr[1], Andreia Brandão[1], Luís Pedro Afonso[1,4], Lúcio Lara Santos[1,2,3,5,6], José Alexandre Ferreira[1,2,6, a]

[1]Research Center of IPO-Porto (CI-IPOP) / RISE@CI-IPOP (Health Research Network), Portuguese Oncology Institute of Porto (IPO-Porto) / Porto Comprehensive Cancer Center (P.ccc) Raquel Seruca, Porto, Portugal; [2]School of Medicine and Biomedical Sciences (ICBAS), University of Porto, Porto, Portugal; [3]School of Medicine and Biomedical Sciences of University Fernando Pessoa, Porto, Portugal; [4]Department of Pathology, Portuguese Oncology Institute of Porto (IPO-Porto), Porto, Portugal; [5]Department of Surgical Oncology, Portuguese Oncology Institute of Porto (IPO-Porto), Porto, Portugal; [6]GlycoMatters Biotech, 4500-162, Espinho.

[a] Corresponding author

José Alexandre Ferreira

Experimental Pathology and Therapeutics Group,

Research Centre, Portuguese Oncology Institute of Porto,

R. Dr. António Bernardino de Almeida 4200-072 Porto,

Portugal; Tel. +351 225084000 (ext. 5111).

Email: jose.a.ferreira@ipoporto.min-saude.pt

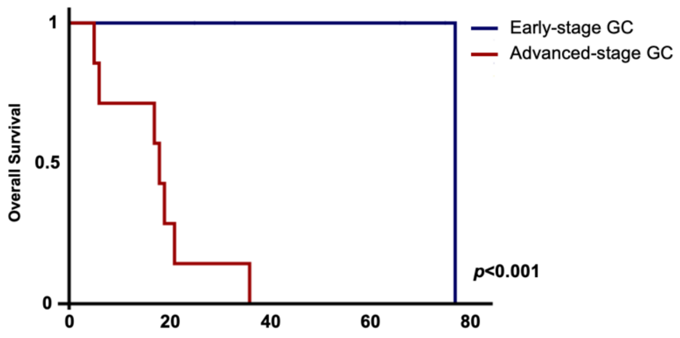

Figure S1. Patients with advanced disease exhibit poorer overall survival (*p* < 0.001). Overall survival was defined as the period between the date of surgery and the date of patient death. Log rank test was used to compare overall survival curves, with statistical significance defined as $p < 0.05$.

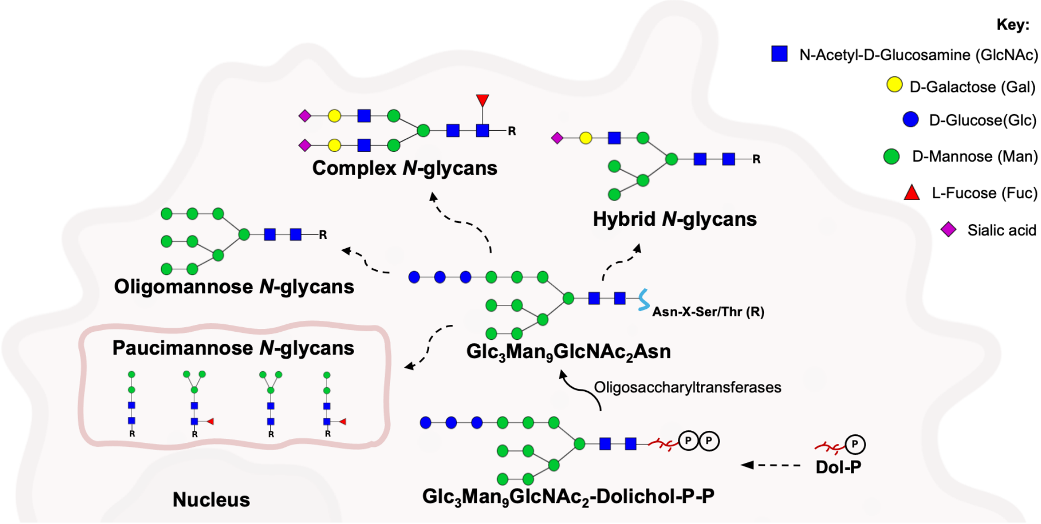

Figure S2. Schematic representation of general pathways of protein-associated *N*-glycan biosynthesis. At the endoplasmic reticulum (ER), the precursor $Glc_3Man_9GlcNAc_2$-P-P-Dol is transferred to asparagine (Asn) in Asn-X-Ser/Thr proteins sequence by oligosaccharyltransferases. Notably, "X" represents any amino acid, except proline (Pro). $Glc_3Man_9GlcNAc_2Asn$ is further processed in the ER and Golgi apparatus given rise to oligomannose, paucimannoses, hybrid and complex *N*-glycans.

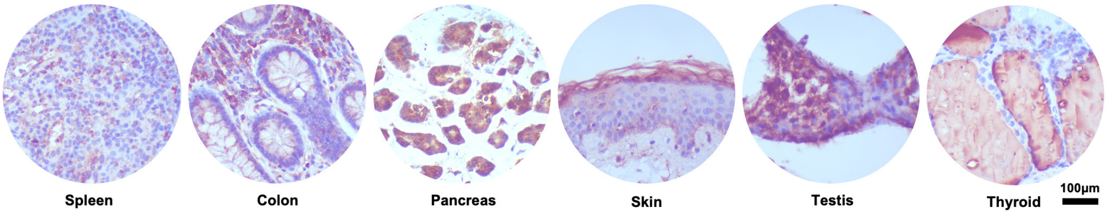

Figure S3. GNL-ligands present a restricted expression pattern in healthy tissues. These are mainly found in pancreas, immune cell populations and testis. Staining was found mainly in the cytoplasm of pancreatic exocrine cells, testicular germ cells, colonic intraepithelial immune cells and in less extension in the basal and keratin layers of epidermis. Staining on thyroid colloid was disregarded.

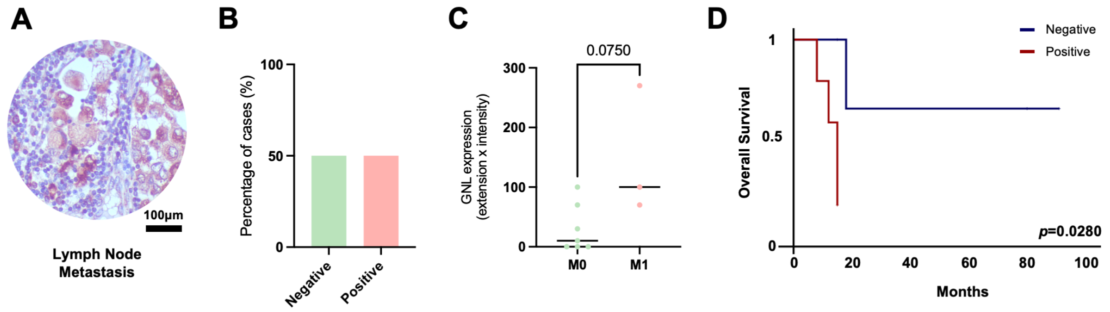

Figure S4. Presence of GNL-ligands on lymph node metastases is a marker of poor prognosis in GC. A and B. GNL staining was primarily observed in the cytoplasm, with a lesser extent on the plasma membrane of tumor cells, in half of the analyzed lymph node metastases (n = 10). **C.** A trend towards overexpression of GNL staining was observed in lymph node metastases of metastatic tumors ($p = 0.0750$). Mann-Whitney test was used to compare GNL expression distribution between non-metastatic (M0) and metastatic tumors (M1), after outliers' removal and data normality determination (Shapiro-Wilk). Significance was considered when $p < 0.05$. **D.** Patients expressing GNL staining in lymph node metastases present worst overall survival ($p = 0.0280$) compared with negative cases in the univariate analysis. Mantel Cox test was used to compare overall survival curves. $P < 0.05$ was set as the threshold for statistical significance.

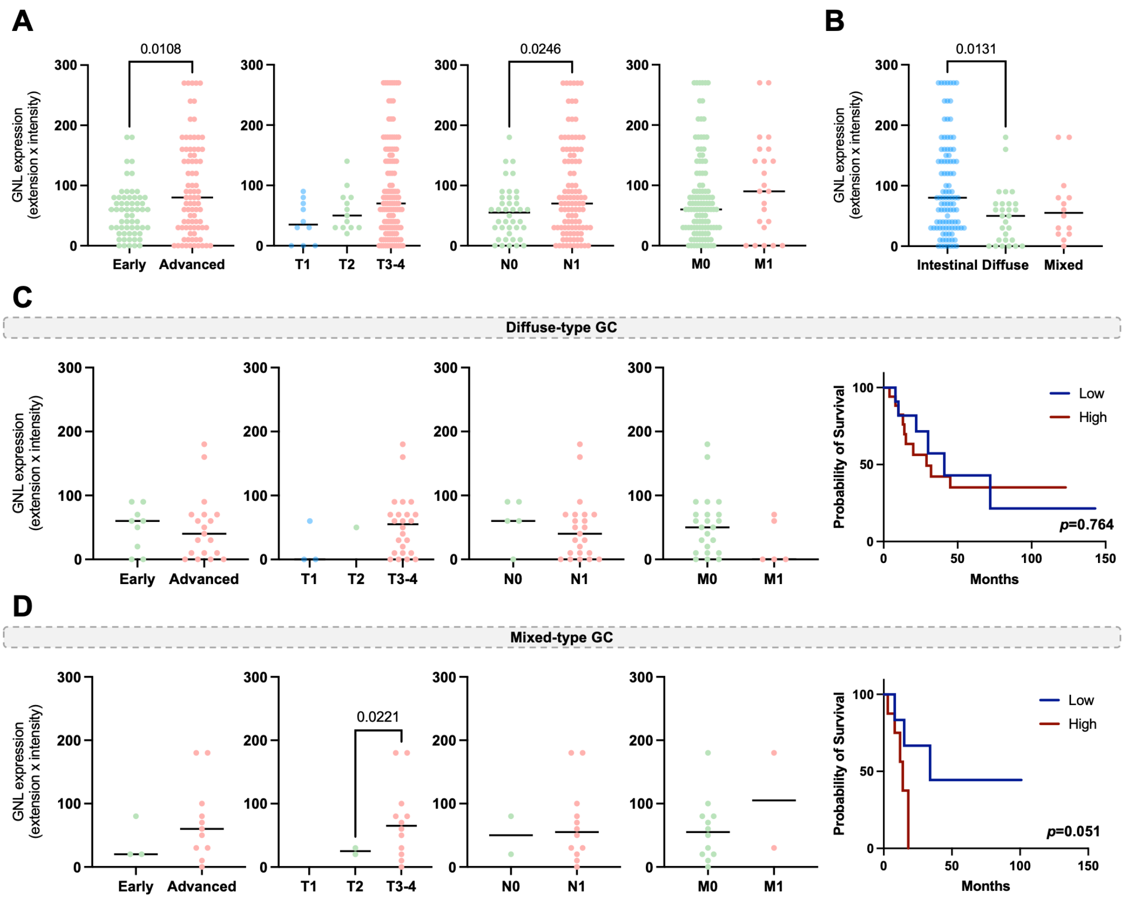

Figure S5. Advanced-stage intestinal-type GC tumours exhibit elevated expression of GNL ligands. A. GC tumours with regional lymph node metastasis exhibit significantly higher levels of GNL ligands. Advanced-stage GC tumours showed significantly higher levels of GNL ligands (*p* = 0.018), with a correlation observed between increased GNL expression in primary tumours and the presence of lymph node metastasis (*p* = 0.0246). No significant association with T stage or distant metastasis was observed (n = 148). Mann-Whitney and Kruskal-Wallis's tests were implemented to assess the distribution of GNL ligand levels across different stages and metastasis statuses. **B. Intestinal-type GC tumours demonstrate higher GNL expression compared to diffuse-type tumours** (n = 144). Kruskal–Wallis' s test was used to compare GNL expression across Lauren's classification groups. **C. The expression of GNL ligands in diffuse-type GC do not correlate with clinicopathological parameters or prognosis (n = 29).** Mann-Whitney and Kruskal-Wallis tests were used to compare groups. Log-rank test was used to compare survival distribution between groups. **D. Advanced T-stage mixed GC tumours present higher levels of GNL.** GNL expression levels were found significantly increased in T3-4 stage tumours (*p* = 0.0221), however no significant differences were observed based on metastasis status (n = 14). A trend towards worse overall survival was noted in mixed GC patients showing overexpression of GNL-ligands (*p* = 0.051). Unpaired t-test with Welch correction was applied to compare variable distribution among groups. The log-rank test was employed to compare overall survival curves. Statistical significance was considered when *p* < 0.05. Statistical testing was preceded by the removal of outliers using the ROUT method (Q = 1%) and an assessment of data normality with the Shapiro-Wilk test.

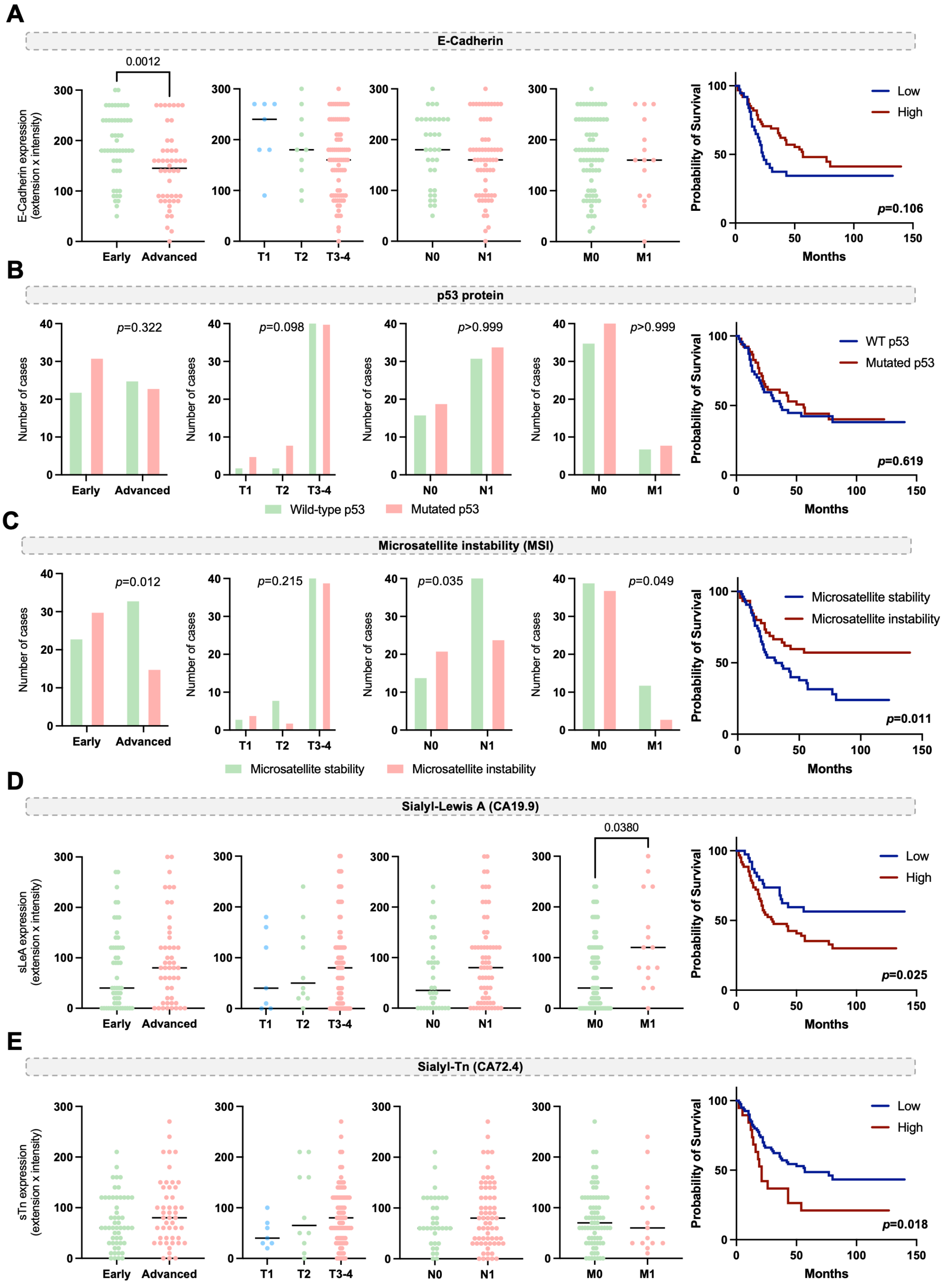

Figure S6. GC classical biomarkers in intestinal-type GC tumours present limited clinical value. A. Advanced-stage GC tumours show reduced levels of E-cadherin, an important protein linked to cell-cell adhesion. E-Cadherin expression was significantly lower in advanced-stage tumors ($p = 0.0012$), but no statistically significant associations were observed across T, N, or M stages (n = 100). Kaplan–Meier survival analysis indicated no significant difference in overall survival between patients with high versus low E-Cadherin expression ($p = 0.106$). Mann-Whitney and Kruskal-Wallis' tests were implemented to

compare E-cadherin levels among groups. Log rank test was used to compare overall survival curves. **B. Mutation status of p53 is not significantly associated with any clinical or pathological variables.** No significant associations were observed across clinical variables, neither in survival (n = 101). Chi-square was used to determine the association between the presence of mutations on p53 and relevant clinicopathological parameters. Differences in patient survival across groups were assessed via log-rank testing. **C. Microsatellite instable tumours are linked to less aggressive traits and better prognosis.** MSI was significant more frequently in early-stage tumours (p = 0.012), especially in non-metastatic tumours (N, p = 0.035; M, p = 0.049; n = 101). Additionally, MSI status was significantly associated with improved overall survival (p = 0.011). Associations between MSI status and clinicopathological parameters were analyzed using the chi-square test. Kaplan–Meier survival analysis with log-rank testing was conducted to compare patient survival across defined groups. **E. Metastatic intestinal-type GC tumours overexpress the Sialyl Lewis A antigen (sLeA), glycosignature linked with poor prognosis.** Significantly elevated sLeA expression was observed in tumours with clinical evidence of distant metastasis (p = 0.038, n = 101). Kaplan–Meier survival analysis revealed a significant association between high sLeA expression and reduced overall survival (p = 0.025). Comparison of sLeA expression levels among groups was performed through Mann-Whitney and Kruskal-Wallis tests. Log rank test was used to compare overall survival curves. **F. Increased levels of sTn are significantly associated with worst prognosis in intestinal-type GC tumours.** Expression of sTn in GC didn´t presented significant differences across clinical stages or TNM classification (n = 101). However, increased levels of sTn were significantly associated with worse overall survival (p = 0.018). To assess differences in sTn distribution between groups, the non-parametric Mann-Whitney test (for pairwise comparisons) and Kruskal-Wallis' test (for multiple group comparisons) were applied. Survival analysis using Kaplan-Meier estimation, followed by log-rank testing, was conducted to compare survival outcomes across the defined patient groups. P values < 0.05 were considered statistically significant. Outliers were removed using the ROUT method (Q = 1%) before statistical testing, and data normality was verified through the Shapiro-Wilk test.

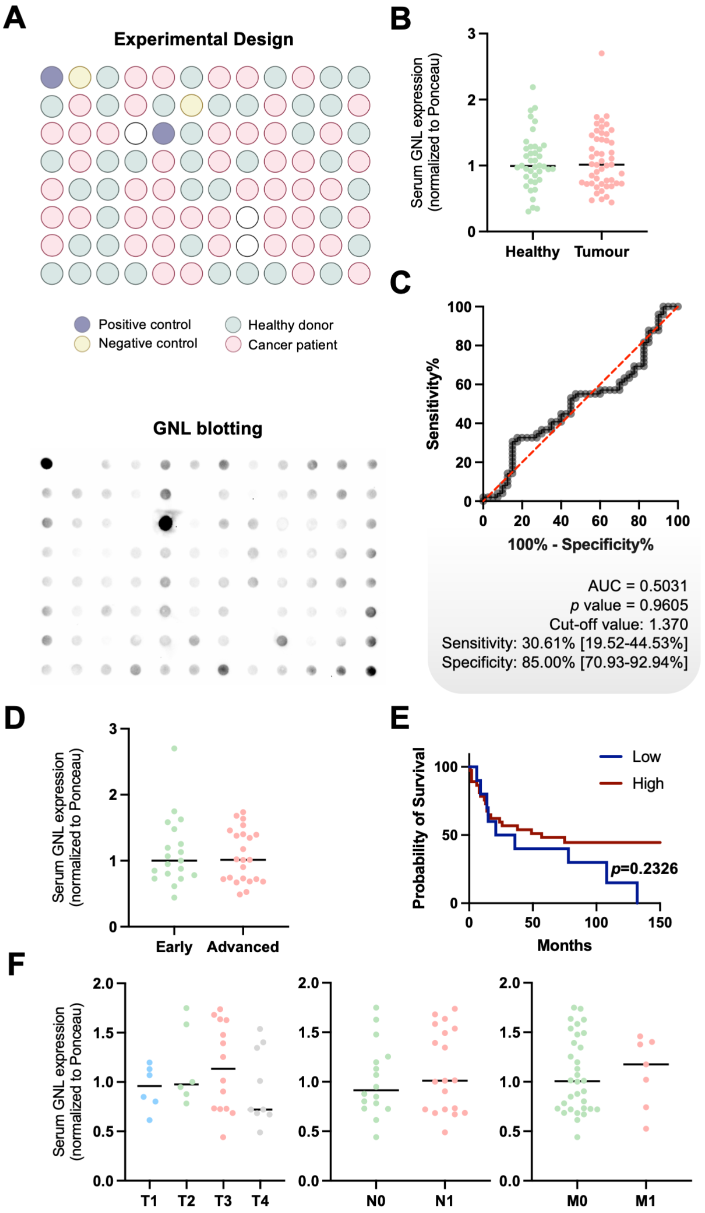

Figure S7. Serum GNL measurement does not exhibit significant discriminatory capability. A. GNL dot blotting reveals no clear differentiation between samples. Blotting for biotinylated GNL unveiled no clear pattern of staining (n = 89). The Dot Blot includes a dispersed arrangement of cancer patient samples (pink, n = 49), healthy donor samples (green, n = 40), and experimental controls (n = 4). Positive controls, consisting of proteins extracted from tumour tissue previously assessed by immunohistochemistry, and negative controls (PNGase F-treated samples or bovine serum albumin), were incorporated into the experimental design. **B. Healthy donors and cancer patients present similar profiles regarding GNL expression in serum.** No significant difference was observed between healthy donors and cancer patients (n = 89). Mann-Whitney test was used to assess differences on GNL levels distribution between healthy donors and cancer patients. **C. Serum GNL levels do not discriminate healthy donors from cancer patients.** ROC curve highlighted no discriminatory power of serum GNL levels, with an AUC of 0.5031 (p = 0.9605). The black ROC curve shows the biomarker's performance, with the red diagonal line indicating the reference for a random classifier. The optimal cut-off value was

determined using Youden's index, and statistical significance was defined as a p value < 0.05. **D, E and F. Serological GNL levels do not correlate with clinicopathological features or prognosis.** No significant correlation was observed between serum GNL levels and clinical stage (D, n = 42), prognosis (E, n = 48, p = 0.2326) or TNM classification (F, n = 40). Mann-Whitney test was used to compare GNL expression among clinical stage (D), N and M stage (F), while Ordinary one-away ANOVA test was used to compare the levels of GNL among T stages (F). Kaplan-Meier survival analysis with log-rank tests was performed to compare patient survival between the defined groups. Optimal cut-off was defined as the optimal discriminate value in a survival ROC curve. Statistical significance was considered when $p < 0.05$. Prior to statistical tests, outliers' removal (ROUT method, Q = 1%) and data normality assessment (Shapiro-Wilk test) were applied.

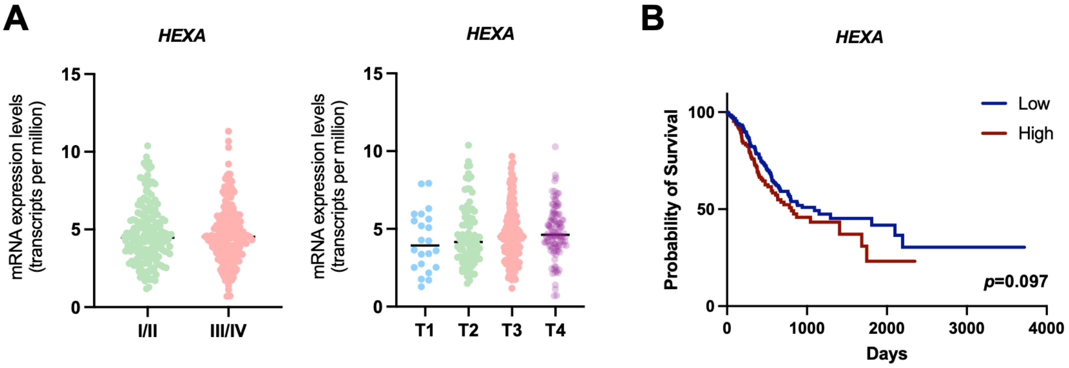

Figure S8. *HEXA* mRNA levels do not change with tumor progression. A. *HEXA* expression (according to TCGA data) does not change with disease progression. Mann-Whitney and Kruskal-Wallis' tests were used to compare the distribution among groups. ROUT method and Shapiro-Wilk tests were performed to remove outliers and determine data normality, respectively. Statistical significance was considered when $p < 0.05$. **B. Tumours exhibiting higher levels of *HEXA* tend to present worst prognosis ($p = 0.097$).** Log rank test was implemented to compare overall survival curves. Results were considered statistically significant if $p < 0.05$.

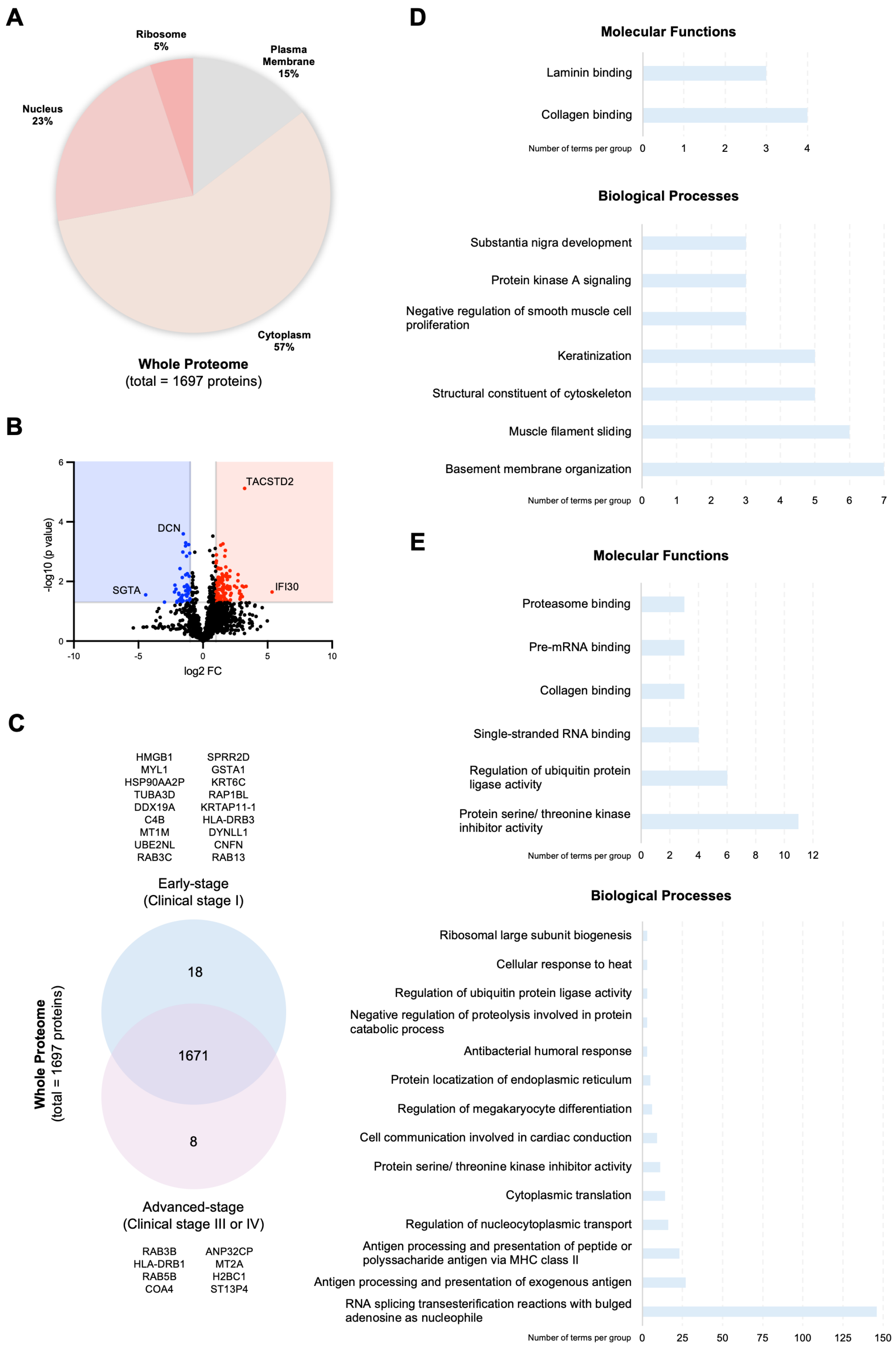

Figure S9. Advanced-stage GC tumors exhibit extensive proteome remodeling. A. Over 1600 proteins were identified across all cell organelles. Identified proteins (n = 1697) were primarily localized to the cytoplasm (57%), nucleus (23%), and plasma membrane (15%) of tumor cells. A small proportion of

ribosomal proteins (5%) were also identified. **B. Scatter plot analysis reveals significant proteomic remodeling with tumor progression.** Advanced-stage tumors overexpress 111 proteins, such as IFI30 and TACSTD2 (red), while early-stage tumors display increased levels of 48 proteins, including SGTA and DCN (blue). **C. Opposing clinical stages exhibit distinct proteomic signatures.** A Venn diagram highlights 8 proteins exclusively expressed in advanced-stage tumors (e.g., RAB3B, HLA-DRB1, and COA4) and 18 proteins unique to early-stage tumors (e.g., HMGB1, MYL1, and C4B). A high degree of homology was also observed, with both stages co-expressing 1671 at variable levels. **D-E. Proteome characterization evidences stage-specific molecular and biological functions.** GO term analysis using Cytoscape highlights that early-stage GC tumors are enriched in proteins involved in extracellular binding, cell adhesion, cytoskeletal remodeling, and substantia nigra development (D). Late-stage tumors are enriched in glycoproteins associated with cellular processes such as RNA splicing and antigen processing and presentation (E). Only pathways with statistical significance ($p < 0.05$) were considered.

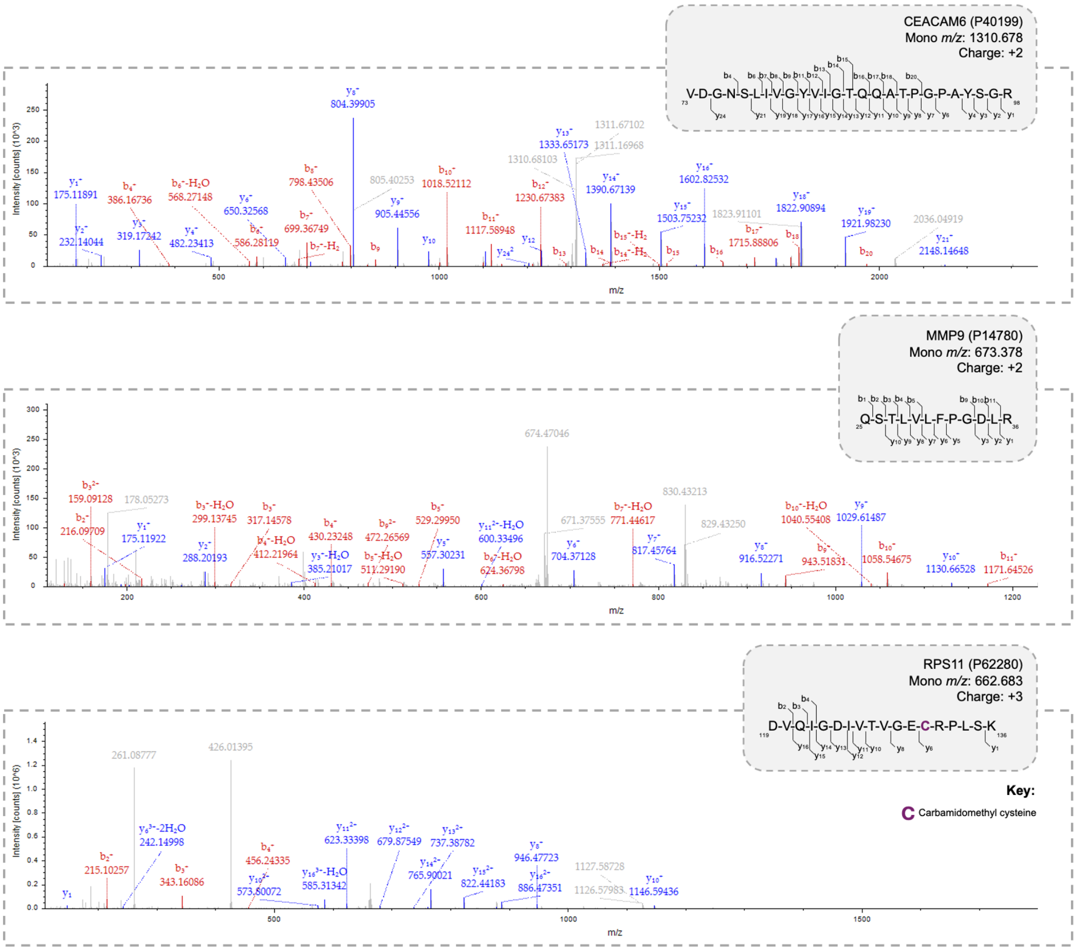

Figure S10. MS/MS spectra support the existence of non-glycosylated peptides of CEACAM6 (P40199), MMP9 (P14780) and RPS11 (P62280) in GC tumour tissues. MS/MS spectra showing well-defined b- and y-ion series, providing high confidence in peptide sequence and protein assignment. The detection and identification of non-glycosylated peptides from CEACAM6, RPS11, and MMP9 further strengthen and validate the identification of these proteins in the glycoproteomic analysis.

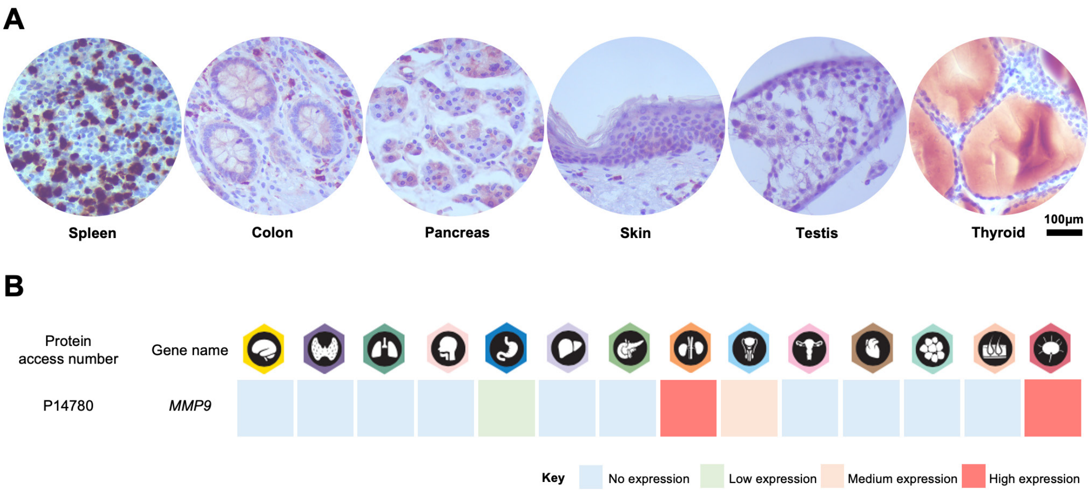

Figure S11. MMP9 expression in healthy tissues is highly restricted to a subset of immune cells. **A. MMP9 expression in healthy tissues was restricted to a subset of immune cells in the spleen and colonic intraepithelial immune cells**. Residual cytoplasmic expression on exocrine pancreas was observed. Our data highlighted no expression of MMP9 in the skin, testis or thyroid. **B. The Human Protein Atlas database supports the existence of limited MMP9 expression in healthy tissues.** MMP9 was particularly evident in the urinary tract (microvilli of proximal tubules), lymphoid organs and testis. Residual expression in mucosal lymphoid cells in colon was also noted.

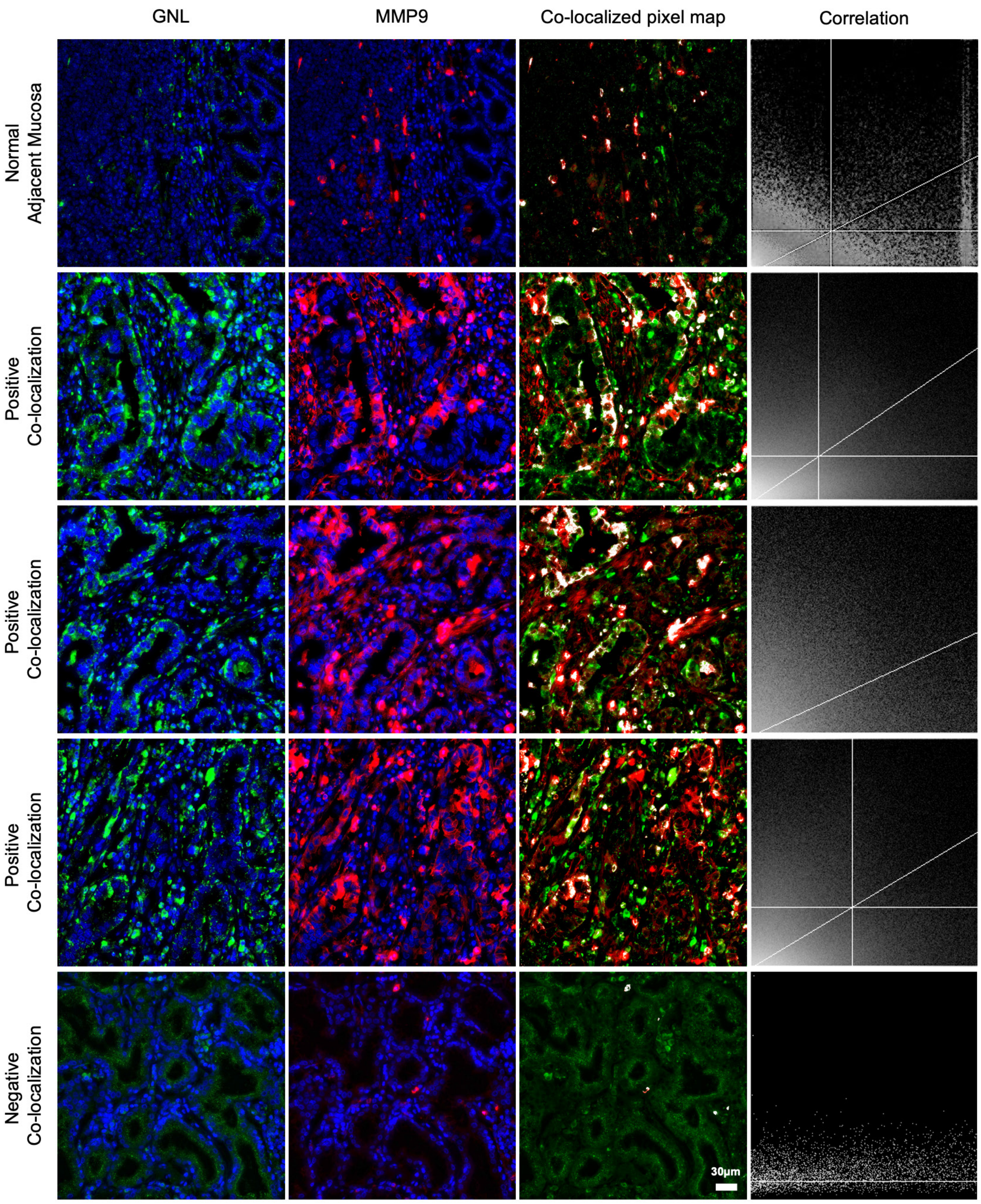

Figure S12. MMP9 and GNL staining is co-localized in GC tumors. Microscopic evaluation of FFPE GC tissues stained for DAPI (blue), MMP9 (red) and GNL (green) unveiled tumor areas with co-localization of MMP9 and GNL (grey/ white), supporting the existence of MMP9 carrying trimmed *N*-glycans (n = 7). This phenotype was not evident on normal adjacent mucosa (first row), neither in areas that are exclusively positive for one of the antigens (fifth row). Additionally, this phenotype appears to be restricted to tumor cells and, to a lesser extent, to tumor secretions. Our data highlight the cancer-associated nature of this glycoproteosignature. Images were acquired using a 40X objective and scale bar = 30 µm. A co-localized pixel map and correlation curve (fourth column) were generated using the "Colocalization Threshold" test in Fiji software.

```
MSLWQPLVLVLLVLGCCFAAPRQRQSTLVLFPGDLRTNLTDRQLAEEYLYRYGYTRVAEMRGESKSLGPALLLLQKQLSL   80
PETGELDSATLKAMRTPRCGVPDLGRFQTFEGDLKWHHHNITYWIQNYSEDLPRAVIDDAFARAFALWSAVTPLTFTRVY  160
SRDADIVIQFGVAEHGDGYPFDGKDGLLAHAFPPGPGIQGDAHFDDDELWSLGKGVVVPTRFGNADGAACHFPFIFEGRS  240
YSACTTDGRSDGLPWCSTTANYDTDDRFGFCPSERLYTQDGNADGKPCQFPFIFQGQSYSACTTDGRSDGYRWCATTANY  320
DRDKLFGFCPTRADSTVMGGNSAGELCVFPFTFLGKEYSTCTSEGRGDGRLWCATTSNFDSDKKWGFCPDQGYSLFLVAA  400
HEFGHALGLDHSSVPEALMYPMYRFTEGPPLHKDDVNGIRHLYGPRPEPEPRPPTTTTPQPTAPPTVCPTGPPTVHPSER  480
PTAGPTGPPSAGPTGPPTAGPSTATTVPLSPVDDACNVNIFDAIAEIGNQLYLFKDGKYWRFSEGRGSRPQGPFLIADKW  560
PALPRKLDSVFEERLSKKLFFFSGRQVWVYTGASVLGPRRLDKLGLGADVAQVTGALRSGRGKMLLFSGRRLWRFDVKAQ  640
                    MVDPRSASEVDRMFPGVPLDTHDVFQYREKAYFCQDRFYWRVSSRSELNQVDQVGYVTYDILQCPED  720
```

Predicted N-glycosites   Covered sequence   Identified glycopeptide

Figure S13. *N*-glycosite prediction unveiled three putative *N*-glycosylation sites on MMP9 protein sequence. According with Net*N*Glyc prediction, MMP9 presents three potential *N*-glycosites on Asn_{38}, Asn_{120} and Asn_{127} (red). Our MS data identified a glycopeptide carrying paucimannosidic *N*-glycans on two predicted *N*-glycosites ($_{116}$WHHHNITYWIQNYSEDLPR$_{134}$; grey).

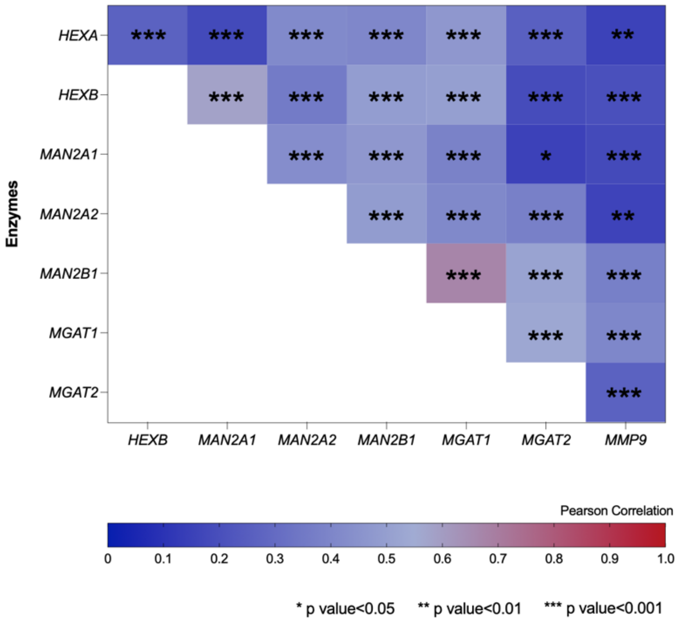

Figure S14. *MMP9* mRNA levels are positively correlated with relevant glycogenes involved in the main biosynthesis route of paucimannosidic *N*-glycans. *MMP9* is correlated in higher degree with *MAN2B1* (R = 0.372*;* $p < 0.001$) and *MGAT1* (R = 0.401*;* $p < 0.001$), and in lower degree with *HEXA* (R = 0.152*;* $p = 0.002$)*, HEXB* (R = 0.199*;* $p < 0.001$)*, MANA21*(R = 1.178*;* $p < 0.001$) and *MAN2A2* (R = 0.157*;* $p = 0.001$) and *MGAT2* (R = 0.264*;* $p < 0.001$) (n = 412). Pearson's correlation method was used to assess the correlation levels between *MMP9*, hexosaminidases (*HEXA* and *HEXB*), mannosidases (*MAN2A1, MAN2A2* and *MAN2B1*) and glycosyltransferases (*MGAT1* and *MGAT2*). A *p*-value of 0.05 was set as minimal requirement for statistical significance.

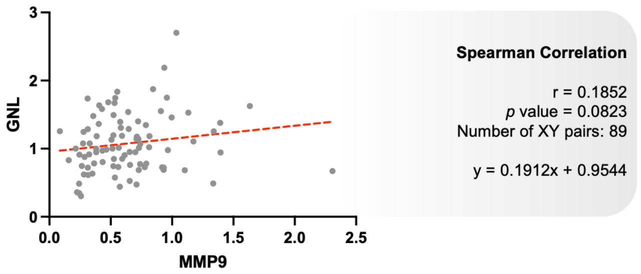

Figure S15. Serological levels of GNL do not correlate with MMP9 levels. Spearman correlation unveiled no significant correlation between serological GNL and MMP9 levels (n = 89; p = 0.0823; R = 0.1852). Statistical significance was considered when $p < 0.05$.
